# Supplementary material for: Nectar-living yeasts of a tropical host plant community: diversity and effects on community-wide floral nectar traits
Source: PeerJ. 2017 Jul 14;5:e3517. doi: 10.7717/peerj.3517 (PMC5511698; doi:10.7717/peerj.3517)
Supplement: Appendix S1 — Number of nectar samples (drops) by host plant species used in the laboratory methods applied in the study. The table shows the sample size for the DNA-based identification of yeasts, the cell count procedures and the HPLC methods for sugar concentration in samples. Total individuals sampled by plant species are also reported. [file peerj-05-3517-s002.docx]

| Host plant species | DNA-identification | Yeast density | Composition and concentration of nectar sugars | Total nectar drops | Total plants |
| --- | --- | --- | --- | --- | --- |
| *Agave angustifolia* | 32 | 32 | 31 | 95 | 10 |
| *Bravaisia berlandieriana* | 23 | 22 | 23 | 68 | 8 |
| *Cordia sebestena* | 18 | 18 | 18 | 54 | 6 |
| *Gossypium hirsutum* | 20 | 20 | 20 | 60 | 7 |
| *Gymnopodium floribundum* | 24 | 24 | 24 | 72 | 6 |
| *Ipomoea crinicalyx* | 25 | 25 | 23 | 73 | 5 |
| *Ipomoea hederifolia* | 22 | 22 | 22 | 66 | 8 |
| *Ipomoea nil* | 40 | 40 | 40 | 120 | 7 |
| *Ipomoea triloba* | 19 | 19 | 15 | 53 | 6 |
| *Lonchocarpus longistylus* | 24 | 24 | 24 | 72 | 8 |
| *Malvaviscus arboreus* | 24 | 24 | 24 | 72 | 9 |
| *Merremia aegyptia* | 37 | 36 | 36 | 119 | 7 |
| *Merremia dissecta* | 21 | 21 | 21 | 63 | 7 |
| *Operculina pinnatifida* | 18 | 18 | 17 | 53 | 6 |
| *Opuntia dillenii* | 29 | 29 | 21 | 79 | 10 |
| *Passiflora foetida* | 16 | 16 | 15 | 47 | 8 |
| *Piscidia piscipula* | 27 | 27 | 27 | 81 | 6 |
| *Tecoma stans* | 20 | 20 | 20 | 60 | 7 |
