# Supplementary material for: Nectar-living yeasts of a tropical host plant community: diversity and effects on community-wide floral nectar traits
Source: PeerJ. 2017 Jul 14;5:e3517. doi: 10.7717/peerj.3517 (PMC5511698; doi:10.7717/peerj.3517)
Supplement: Supplemental Information 1 — Nucleotide sequences of the large-subunit (26S) ribosomal DNA gene (D1/D2 region). Each sequence was obtained by two-way sequencing method using the primer combination NL1-NL4, according to Kurtzman & Robnett (1998) and Lachance et al. (1999). Raw sequences were edited, assembled and consensus sequences were obtained using Geneious Pro 8.1.7 bioinformatics software (Biomatters Ltd, Auckland, New Zealand). [file peerj-05-3517-s003.docx]

>Seq1 [organism=Candida sorbosivorans] [19CF_isolated from floral nectar of Agave angustifolia (Agavaceae)] D1/D2 LSU rDNA gene partial sequence

CAAGTGTCTCTGTTGCGAAGAGAAACGTGAGAAAGGCTAAGAAAGGCGAAATTCTTATTTCCAACCCTCGGGGTTGTAATCTGGATACCTGGATTTGGCACCCTGATAAGTCTTCTGGAACGGACCGCCATGGAGGGTGACAGCCCCGTAGCAGCAGCCACTGTAAATCCGGGTCGACGAGTCTAGTTGTTTGGGAATGCAGCTCTTGTGGGTGGTATGCTCCATCTAAAGCTAAATATTGGCGAGAGACCGATAGCAAACAAGTACTGTGAAGGAAAGATGAAAAGAACTTTGAAAAGAGAGTGAAAAAGTACGTGAAATTGTTGAAATGGAAGGCAATGAGGTGCGATTGAACCGGTTTTTTGTGGGCAGGACAAAAGCGCAGGCCGCCTCGGCATTGCCTGCGTGCATACTGCCTCGCGGACTACCGGTTCTAACAACGCCTTATTGCACCCCTCTTGAAACACAGACCCCGGGTTATATTATTCCGGAGAAAATTTCCCCCCCCCTTAAAAAAAAAGCGGGGGGAGTATTTGTAGAGAAAGAAAACCTCCCCCCGAGGGTTAGAAGAAAGGGGGACATATACATCAGAGAAGAAGGTGATGTGAACACAAAAAAGGAGTGAAGTCGCTAAGAGGAAAGAGTGAACCCATCGGCGTCTCCCCATAGTAGGAGACCCTCCAGGTCGGCCGCACCCGATAATTCGTCCATATCCTAACCGCACGCGGCTATAGGCAAGCAACAGCTGCCCCAGAACACAAGAGAGAATACGAAGAAACAAATGAAGAAGGGGAGAGATGCGTAGATAGAACGAGAGAAAAGGACGGCGACAGATAACGAAATAGTAATGCAGAAAGGATGCATATGGGATGCAAGGAGCATGGGAGCAATAGATGATAAACTGATCAGACGATGCATAAAAAGAGAAACAAAAGAGAGTGTACAGGGACCGTCCTGGCGCACACGATCACCAAATAGAGACTAAGCAAAGCCCGCACACGACGGAACTGAGGCACAAACATGACTGCATAAGTCAGTAAGGGCTAGCAGGGGGGGCTAAATGG

>Seq2 [organism=Clavispora lusitaniae] [16CF_isolated from floral nectar of Agave angustifolia (Agavaceae)] D1/D2 LSU rDNA gene partial sequence

CCTGCGGGAATTGTAATTTGAAGGTTTCGTGGTCTGAGTCGGCCGCGCCCAAGTCCATTGGAACATGGCGCCTGGGAGGGTGAGAGCCCCGTCCGGCGCACGCCGACTCTTTGCACCGCGGCTCCGACGAGTCGAGTTGTTTGGGAATGCAGCTCTAAGTGGGTGGTAAATTCCATCTAAAGCTAAATATTGGCGAGAGACCGATAGCGAACAAGTACAGTGATGGAAAGATGAAAAGCACTTTGAAAAGAGAGTGAAACAGCACGTGAAATTGTTGAAAGGGAAGGGCTTGCAAGCAGACACGGTTTACCGGGCCAGCGTCGAAAAGGGGGGAGGAACAAGAACTCGAGAATGTGGCGCGC

>Seq3 [organism=Hannaella siamensis] [7CF_isolated from floral nectar of Agave angustifolia (Agavaceae)] D1/D2 LSU rDNA gene partial sequence

TTACCTCTCCCCTAGTAGAGGCTGTAGCTGAACCAAGGTGTGTTTACCTTGGTAAGCTCAAATTCCCAATAAAATCGACGAATGCGTCTTTCGAAGGCGTGGCCGAGCTGTAATCTACACAAGTGTTTTTCCGTGCCGGACCATGTCCAAGTCCCTCGGTAAAAGGGTATCCCAGCCTAGGGGTCACAATCCCGTACTTGACACGACAACCGGTGCTCTATGATACACCTTTCTACGAATCGAGTCGTTGGGGAATAAAAAAAAAAAAAGAGTGGTAAATTCCATCTAAAGCTATACATATACGCAAAAGACCGATAGCGAACAAGTACCGTGAGGGAAAAATAAAAAGCACTTTGGAAAGAGAGTTAAACAGTACGTGAAATTGTTGAAACGGAAACGATTGAAGTCAGTCGTGACTGTTGGTCTAAGCTGGTTCTGCCAGTGTATTACCAGCATTCGGGCCAAGCATCAGTAATTGAGCGGAGGAAAAAGGTATGGAGAACGTAGCACCTTCGGGTGTGTTATAGCTCCATATTGCATACACTGCTTGAGACTGAGGAATGCACCTCGCCTGTATGGCCAGGGTTCGCCCACTTCCGAGCTTAGGATGTCGACCATAGATGGCTTTAAACAACCCGTCGTGAGCAA

>Seq4 [organism=Papiliotrema flavescens] [4CF_isolated from floral nectar of Bravaisia berlandieriana (Acanthaceae)] D1/D2 rDNA gene partial sequence

CAAGTGATCCCCTCTCAAGTGCTTGATACGGTCCCACGGCAAGTGAACCGGGAAAAGCTCAAATTTCAAATCTGGCGTGCTCAGTGCGTCCGAGTTGTAATCTAAAGAGGCGTTTTCCGTGCCGGACTGTGTCCAAGTCCCTTGGAACAGGGTATCAAAGAGGGTGATAATCCCGTACTTGACACAATGACCGGTGCTCTGTGATAAGTCTTCTACGAGTCGAGTTGTTTGGGAAAGCAGCTCAAAAAGGGTGGTGAGTTCCATCTAAAGCTAAATATTGGCGAGAGACCGATAGCGAACAAGTACCGTGAGGGAAAGATGAAAAGCACTTTGGAAAGAGAGTTAAACAGTACGTGAAATTGTTAAAAGGGAAACGATTGAAGTCAGTCGTGAATGAGAGGCTCAGCCGGTTCTGCCGGTGTATTCCCCTCAGTCGGGTCAACATCAGTTTTGTTCGGTGGATAAGGGCAGCTGGAAGGTAGCACCCTCGGGTGTGTTATAGCCAGTTGTCGCATACATCGGATGAGACTGAGGAATGCAGCTCGCCGGGATGGCCGGGGTTAGCCCACGTTCGAGCTTAGGAGGTTCCCAGACATAATGGCTTTAAACGACCCGTAGTGAGAACA

>Seq5 [organism=Cryptococcus sp. 1] [11CF_isolated from floral nectar of Bravaisia berlandieriana (Acanthaceae)] D1/D2 LSU rDNA gene partial sequence

CCAGGACTCCCCTCTAGCACATCGGAACGTTACAACGGCGAGTGAACCGGGATGAACAAAAATTTAAAATCGGGCGTCCTTCGGGCGTCCGACACGTTGTAATCTAAGAGGGCGTTTTCCGCGCCGGACCGCGTCCAAGTCCCTTGGAATAGGGTATCAAAGAGGGTGACAATCCCGTACTTGACGCGACGACCGGTGCTCTGTGATACGTTCTCAACGAGTCGAGTTGTTTGGGAATGCAGCTCTAAATGGGTGGTAAATTCCATCTAAGGCTAAATATTGGCAAGAGACCGATAGCGAACAAGTACCCTGAGGGAAAGATGAAAAGCACTTTGGAAAGAGAGTTAAACAGCACGTGAAATTGTTGAAAGGGAAACGATTGAAGTCAGTCGTGTGGAGGGTATTCAGCCGTCTCTGGCGGTGTACTTGCCCTTCACGGGTCAACATCAGTTTGATCCGGTGGAAAAAGGCTGGGGGAAGGTGGCACCCCCGGGTGTGTTATAGCCCCCTGTCACATACACCGGACCAGACTGAGGAACGCACCTCGCAGCAATGCCGGGATTCGCCCACGTACGAGCTTAGGATGTTGACAAGAACAACTTAAAACGACCCGTCTTGAGGAA

>Seq6 [organism=Candida apicola] [65CF_isolated from floral nectar of Cordia sebestena (Boraginaceae)] D1/D2 LSU rDNA gene partial sequence

AAACCAACAGGGATTGCCTTAGTAGCGGCGAGTGAACAGGCAAGAGCTCAGATTTGAAAGCCCTCGGGCATTGTATTCTGAAGCCTTGGTCCTGAGAATCGGTGTTTAAGTCTTCTGGAAAGGAGCGCCATGGAGGGTGATAGCCCCGTACGACACCACTCTCATTGTAGGACTTTGGCATGGAGTCGAGTTGTTTGGGAATGCAGCTCAAATGGGTGGTATGCTCCATCTAAAGCTAAATATCTGCGAGAGACCGATAGCGAACAAGTACTGTGAAGGAAAGATGAAAAGAACTTTGAAAAGAGAGTGAAAAAGTACGTGAAATTGTTGAAATGGAAGGATAGGCCGCTAACCATGTAGGGTCGTGTTCGAGGGGAAGATAAAAGCTGAAGAATGTAGCTCCTCGGAGTGTTATAGCTTCAGTCCATATTCCCTCTCGAGCGCGAGGATTGAAGACTCTGCTAAATGGTGGTCTATCAC

>Seq7 [organism=Candida versatilis] [61CF_isolated from floral nectar of Gossypium hirsutum (Malvaceae)] D1/D2 LSU rDNA gene partial sequence

AAACCAACCGGGATTGCCCTAGTAACGGCGAGTGAAGCGGCCAAAGCCCAAATTTGAAATCCTCTTTTGAGGAGTTGTAATTTGGAGATGGAGTGCTGGACTCAGCTCTGTGGAAGTTGGCTGGAAAGCCGCGCCTTGGAGGGTGATAGCCCCGTGCCACGGAGGTCCTGTGTCTGTGTAGTACTCTTTCTACGAGTCGCGTTGTTTGGGAATGCAGCGCTAAGTGGGTGGTAAATTCCATCTAAGGCTAAATATTGGCGAGAGACCGATAGCGAACAAGTACTGTAAAGGAAAGATGAAAAGCACTTTGAAAAGAGAGTGAAATAGCACGTGAAATCGTTGAAGTGGAAGGGCTTAAAGCTACACACCTTCTTCGGAAGGGGCTAACATCAGTTTTGCCGGATGGATAAATGGTAAAGAAAGTGGCATCTTCGGATGTGTTATAGCTTTATCAAATACGTCCCGGCGGGATTGAGGACCGCGCTTTATGCAAGGATGTGGGCGTAAGGGTTTTAACCAAC

>Seq8 [organism=Vishniacozyma taibaiensis] [53CF_isolated from floral nectar of Gossypium hirsutum (Malvaceae)] D1/D2 LSU rDNA gene partial sequence

AAACTAACTAGGATTCCCTTAGTAACGGCGAGTGAACCGGGATGAGCTCAAATTTAAAATCTGGCGTCCTTCGGGCGTCCGAGTTGTAATCTATAGAGGCGTTTTCCGCGCCGGACCGCGTCCAAGTCCCTTGGAATAGGGTATCAAAGAGGGTGACAATCCCGTACTTGACGCGACGACCGGTGCTCTGTGATACGTTCTCAACGAGTCGAGTTGTTTGGGAATGCAGCTCTAAATGGGTGGTAAATTCCATCTAAGGCTAAATATTGGCGAGAGACCGATAGCGAACAAGTACCGTGAGGGAAAGATGAAAAGCACTTTGGAAAGAGAGTTAAACAGCACGTGAAATTGTTGAAAGGGAAACGATTGAAGTCAGTCGTGTGGAGGGTATTCAGCCGTCTCTGGCGGTGTACTTGCCCTTCACGGGTCAACATCAGTTTGATCCGGTGGAAAAAGGCTGGGGGAAGGTGGCACCCTCGGGTGTGTTATAGCCCCCTGTCACATACACCGGACCAGACTGAGGAACGCAGCTCGCAGCAATGCCGGGGTTCGCCCACGTACGAGCTTAGGATGTTGACATAATGGCTTTAAACGAC

>Seq9 [organism=Starmerella bombicola] [55CF_isolated from floral nectar of Gossypium hirsutum (Malvaceae)] D1/D2 LSU rDNA gene partial sequence

AAACCAACAGGGATTGCCCTAGTAACGGCGAGTGAACAGGCAAAAGCTCAGATTTGAAAGCCTCTCGGGGCATTGTATTCTGAAGCCTTGATTCTGAGAACCGGTGCCTAAGTCTTCTGGAAAGGAGCGCCATGGAGGGTGATAGCCCCGTACGGCACTGACCTCATTGTAGAATCTTGGCGTGGAGTCGAGTTGTTTGGGAATGCAGCTCAAATGGGTGGTATGCTCCATCTAAAGCTAAATATCTGCGAGAGACCGATAGCGAACAAGTACTGTGAAGGAAAGATGAAAAGAACTTTGAAAAGAGAGTGAAAAAGTACGTGAAATTGTTGAAATGGAAGGATAGGCCGCTAACCACGTAGAGCCGTGTCTGAGGGGAGGATAAAAGCTGTAGAATGTGGCTCTTCGGAGTGTTATAGCTACAGTGCATACTCCCACTCGGGCGCGAGGACTAAGGCGCTCTGCTAAATGGTGGTCTATCAC

>Seq10 [organism=Sympodiomycopsis paphiopedili] [63CF_isolated from floral nectar of Gossypium hirsutum (Malvaceae)] D1/D2 LSU rDNA gene partial sequence

AAACTAACAAGGATTCCCCTAGTAACGGCGAGTGAAGCGGGAAGAGCTCAAATTTGAAAGCTGGTACCTTCGGTGCCCGCGTTGTAATCTCGAGAAGTGTTTTCCGTGCTGGACCATGTACAAGTTCCTTGGAATAGGACGTCATAGAGGGTGAAAATCCCGTACTTGACATGGATGCCCAGTGCTTTGTGATACACTCTCCACGAGTCGAGTTGTTTGGGAATGCAGCTCAAAATGGGTGGTAAATTCCATCTAAAGCTAAATATTGGGGAGAGACCGATAGCGAACAAGTACCGTGAGGGAAAGATGAAAAGCACTTTGGAAAGAGAGTTAAACAGTACGTGAAATTGTCGAAAGGGAAGCGCTTAAAGTTAGACATGCCTATTGGGATTCAGCCTTGCTTTTGCTTGGTGTATTTCCCGGTGAGCAGGCCAGCATCAGTTTCGGCTGTCGGATAAGGGTTAGAGGAATGTGGCCCCTCGGGGTGTTATAGCCTTTAACTGGATACGGCGGCTGGGACTGAGGAACGCAGCGTGCCTTTATGGCGGGCCTTCGGGCACCTTCACGCTTAGGATGCTGGCGTAATGGCTTTAAGCGAC

>Seq11 [organism=Candida sorbosivorans] [41CF_isolated from floral nectar of Gymnopodium floribundum (Polygonaceae)] D1/D2 LSU rDNA gene partial sequence

CGCGGCCTGCTCTTTTGTCCTGCCCGCTGACGGCCGGTCCCCGCGCACGGTGACGCCCTGGTAGCAACGGCGACTGTCCATCCGGGTCGACGAGTCGAGTTGTTTGGGAATGCATCTCTTACTGGTTGGTTTGCTCGGTCTTACGCTTAATATTGGCTAGTGACTTATAGCTAACACTTACTGTGAACGAAAGATCAAAAGAACTTTGATACCATAGTGAAAAAGTACGTGAAATTGTTGAAATGGAAGGCAATGAGGTGCGATTGAACCGAACGTTTGCCGACAGGACTCAATCGCAGGCCGCCTGCGCATTGCCTGCGTGCATACTGCCCCCCGGACTCCCGGTTCTAACAACGCCGTATTGCAC

>Seq12 [organism=Papiliotrema laurentii] [40CF_isolated from floral nectar of Gymnopodium floribundum (Polygonaceae)] D1/D2 LSU rDNA gene partial sequence

ACTAACAAGGACCTCCCCTACTAACGGCGAGCGAACCGGGAAGAGCTCAAACCTGAAACCTGGCGTCCTCAGGGCGGCCGAGCCGTAATCTATAGAGGCGCTTTCCCTGCCGGACCGGGTCCAAGCTCCTTGGAACAGGATATCAAAGACGGTGACAATCCCGTACTTGACACGACGACCGGTGCTCTGTGATACGTCTTCTAACCAGTCGAGTTGTTTGGGAATGCACCTCAAAATGGCAGGTGAGTTCCATCTAAAGCTAAATATTGGCGAGAGACCAATAGCAAACAAATACCGTGAGGGAAAGATGAAAAGCACTTTGGAAAGAGAGTTAAACAATACGGGAAATTGTTGAAAGGGAAATCCATTGAAGTCAGTCGTGACCGACAGGCTCAGCCGGCGTCTGCCGGTGTATTCCCCTCGGTCGGGTCAACATCAATTTTGTCCGGTGGATAAGGACGGCAGGAAGGTGGCACCCTCGGGTGTGTTATAGCCTGCCGTCGCATACATCGGGTGAGACTGAGGAACGCAGCTCGCCGGTATGGCCGAGGTTCGCCCACCTCCGAGGCTAACGATGTTGACATAATACATCGCTGATGT

>Seq13 [organism=Papiliotrema flavescens] [39CF_isolated from floral nectar of Gymnopodium floribundum (Polygonaceae)] D1/D2 LSU rDNA gene partial sequence

AAACTAACAAGGATTCCCCTAGTAACGGCGAGTGAACCGGGAAGAGCTCAAATTTGAAATCTGGCGTGCTCAGTGCGTCCGAGTTGTAATCTATAGAAACGTTTTCCGTGCCGGACCGTGTCCAAGTCCCTTGGAACAGGGTATCAAAGAGGGTGATAATCCCGTACTTGACACGACGACCGGTGCTCTGTGATACGTTCTCTACGAGTCGAGTTGTTTGGGAATGCAGCTCAAAATGGGTGGTGAGTTCCATCTAAAGCTAAATATTGGCGAGAGACCGATAGCGAACAAGTACCGTGAGGGAAAGATGAAAAGCACTTTGGAAAGAGAGTTAAACAGTACGTGAAATTGTTGAAAGGGAAACGATTGAAGTCAGTCGTGACTGAGAGGCTCAGCCGGTTCTGCCGGTGTATTCCCCTCAGTCGGGTCAACATCAGTTTTGTCCGGTGGATAAGGGCAGTTGGAAGGTGGCACCCTCGGGTGTGTTATAGCCAGCTGTCGCATACATTGGATGAGACTGAGGAATGCAGCTCGCCTTTATGGCCGGGGTTCGCCCACGTTCGAGCTTAGGATGTTGACGTAATGGCTTTAAACGAC

>Seq14 [organism=Candida etchellsii] [313CF_isolated from floral nectar of Ipomoea crinicalyx (Convolvulaceae)] D1/D2 LSU rDNA gene partial sequence

ATTCGAGGGTATTGCCCTAGTAACGGCGAGTGAACAGGCAAAAGCTCAGATTTGAAAGCCTTTTTTGGCATTGTATTCTGAAGTCAGTTTCCTGAGTATCGATGTTTAAGTCTTCTGGAAAGGAGCGCCATGGAGGGTGATAGCCCCGTACAACATCACACTCATTGTAGGAGTCTGACGTGGAGTCGAGTTGTTTGGGAATGCAGCTCAAAAGGGTGGTATGCTCCATCTAAAGCTAAATATCTGTGAGAGACCGATAGCGAACAAGTACCGTGAGGGAAAGATGAAAAGAACTTTGAAAAGAGAGTGAAATAGTACGTGAAATTGTTGAAATGGAAGGGTAGGCCGCTAACCATGTAGAACCGTGTCTGGGGGGAGGACAAAAGCTGAAGAATGTAGCTCCTCGGAGTGTTATAGCTTCAGTCAATACTCCCAGGCCGGGCGCGAGCACCACCAGTGCTTATGACAGAGAATAGATGCG

>Seq15 [organism=Candida powellii] [303CF_isolated from floral nectar of Ipomoea crinicalyx (Convolvulaceae)] D1/D2 LSU rDNA gene partial sequence

AACTACAGGGTATTGCCTTAGTAGCGGCGAGTGAATAGGTATAGAGCTCAGACCTGAAAGCCTTTCGGGGCATTGTATTCTGAAGCCTTGGTCTCGGGAATCGGTGCTTAAGTCTTCTGGAAAGGAGCGCCATGGAGGGTGATAGCCCCGTGAAGCACTAACCCCATTTTGGGACCTTGGCGTGGAGTCGAGTTGTTTGGGAATGCAGCTCAAATGGGTGGTATGCTCCATCTAAAGCTAAATATCTGCGAGAGACCGATAGCGAACAAGTACTGTGAAGGAAAGATGAAAAGAACTTTGAAAAGAGAGTGAAATAGTACGTGAAATTGTTGAAATGGAAGGGTAGGCCGCTAACCACGTAGAGTCGTGTCTAGGGGGAGGATAAAAGCTGCAACCTATATCATTCTTCGGAGTGGTATAGGCGCGGTGCATACTCCCACCCGGGCGCGAGGACCTAAGACTCTACTAAATGGTGGTCTACCACCCGTCTTGAAACCGGGACCAAAGGGCATTGGATTCTGAAGCCTTGGTCTCGGGAATCGGTGCTTAAGTCTTCTGGAAAGGAGCGCCTTGGAGGGTGATAGCCCCGTGAAGCACTCACCCCATTTTGGGACCTTGGCGTGCCGTCCAGTTGTTTGGGAATGCAAATAAAATGGGTCGTATGCTCCATCTAAAGCGAAAAACTCTGCGAGAGACCGAGCGCGCAAAAAGTTCTGTGGATGAAAAGATGCAAAAGACATTTGCTAATAGAGCTGTGATAGTTCTTTCAATGATTGCATAGCAAAGATAGCCGTTTTCCAACGTAATGTACTGTCTGGTGTGGGATAAAACTGCACCATTATAGTTTGCTCGCATGCGTATGGAACCAGCAATGCTACCAAACGGGACAATGACTTCGTAACGTGATACACCCTCTCGTATA

>Seq16 [organism=Metschnikowia ipomoeae] [320CF_isolated from floral nectar of Ipomoea crinicalyx (Convolvulaceae)] D1/D2 LSU rDNA gene partial sequence

ACTGAGGGCATTACCTTAGTAACGGCGAGTGAAGCGGCAAAAGCTCAAATTTGAAATCTTCGGAATTGTAATTTGATGGATAAAATTGCCACGGACAAAAGTTCACTGGAAAGTGACGCCGTAGAGGGTGATAGCCCCGTTTACGTCCACCGGCACTATAATTTTATTCCAAAGAGTCGGGTTGTTTGGGAATGCAGCTCAAAGTGGGTGGTAAATTCCATCTAAAGCTAAATATTGACGAGAGACCGATAGCGAACAAGTACAGTGATGGAAAGATGAAAAGAACTTTGAAAAGAGAGTGAAACAGTACGTGAAATTGTTGAAAGGGAAGGGGAGGGAAGATGTTAAAGGGAACTCCTTCTTTAGTAGGGAGCCCCACCAAGAGTCTATCCTCTCGGG

>Seq17 [organism=Metschnikowia lochheadii] [304CF_isolated from floral nectar of Ipomoea crinicalyx (Convolvulaceae)] D1/D2 LSU rDNA gene partial sequence

CTCCCCTTCCCTTTCAACAATTTCACGTACTGTTTCACTCTCTTTTCAAAGTTCTTTTCATCTTTCCATCACTGTACTTGTTCGCTATCGGTCTCTCGTCAATATTTAGCTTTAGATGGAATTTACCACCCACTTTGAGCTGCATTCCCAAACAACCCGACTCTTTGGAATAAAATTATAGTGCCGGTGGACGTAAACGGGGCTATCACCCTCTACGGCGTCACTTTCCAGTGAACTTTTGTCCGTGGCA

>Seq18 [organism=Metschnikowia sp.] [310CF_isolated from floral nectar of Ipomoea crinicalyx (Convolvulaceae)] D1/D2 LSU rDNA gene partial sequence

ATACGAGGGTATTGCCTCAGTAACGGCGAGTGAAGCGGCAAAAGCTCAAATTTGAAATCCCCCGGGAATTGTAATTTGATGGTGGGTCGATGGGAGTTTAGGAAAGTTTACTGGAAAGTAACGCTATAAAGGGTGACAGCCCCGTTCCTTTTTCCCCCCCGACCCTCCTAAGAGTCGAGTTGTTTGGGAATGCAGCTCTAATGGTGGTAAAATCCATCAAAAGCTAAATATCAGCGAGAGACCGATAGCGAACAAGTACAGTGATGGAAAGATGAAAAGCACTTTGAAAAGAGAGTGAAAAAGTACGTGAAATTGTTGGAAGGGAAGGGATTATTGGGTAGCGAGGTGCCAGGTGGGGAAGAAAGAAAAATCGGAAGGAATGTGGCTCCTCGGAGTGTTATATCCTACCGCCAAATTTCTATTCCCGCATAAGGCCCGCTTTATAGGGCCCCACCAGAAGCTCAAGAATTCACCCGTCTTG

>Seq19 [organism=Starmerella sp.] [337CF_isolated from floral nectar of Ipomoea crinicalyx (Convolvulaceae)] D1/D2 LSU rDNA gene partial sequence

ACTAGAGGGCTTGCCTTAGTAGCGGCGAGTGAACAGGCAAAAGCTCAGATTTGAAAGCCTCTCGGGGCATTGTATTCTGAAGTCAGTTTCCTGAGTGTCGATACTTAAGTCTTCTGGAAAGGAGCGCCATGGAGGGTGACAGCCCCGTACTGTATCTAACTCATTGTAGGAGTCTGACGTGGAGTCGAGTTGTTTGGGAATGCAGCTCAAAAGGGTGGTATGCTCCATCTAAAGCTAAATATCTGTGAGAGACCGATAGCGAACAAGTACTGTGAAGGAAAGATGAAAAGAACTTTGAAAAGAGAGTGAAATAGTACGTGAAATTGTTGAAATGGAAGGGTAGGCCGCTAACAATGTAGAACCGTATTCGGGGGGAAGATAAAATCTGTTGAATGTGGCTCTTCGGAGTGTTATAGCTTCAGACAATATTCCCACCTGGGTGCGAGGATCTCAGGTTCTACTAAATGGTGGTCTACCACCCGTCTTGAAACCGGGACCAAAAGTCGATAGATGTACGCGGTCCAGGGGGGTGGTTTATTAAGACTGTTCATAGTTTTTATAGTGAATCTTCTTGCTAGGCGGCCATAAAAAGGAGAACCCTGACTTTTTTCTAGAAAAGTAGAGAGAAAAACGGCGGCCACCAACGTAATATCCCTAA

>Seq20 [organism=Wickerhamiella occidentalis] [341CF_isolated from floral nectar of Ipomoea crinicalyx (Convolvulaceae)] D1/D2 LSU rDNA gene partial sequence

AACATGAGGACTTGCCTTAGTAGTGGCGAATGAACCGGCAAAAGTCCAAATTTGAAATCTGCCAGCAATGGCCGAGTTGTAATTTGAAGATGGTAATTCTGTAGAGAGTCTTTGCTCAAGTTTCCTGGAATGGAACGCCGTGGAGGGTGACAGCCCCGTGAGGCATTGACTTAATACGTGTAGAGTACTATCAAAAAGTAAAATAGTTTGGGAATGCAGCTCAAATCTCGTGGTAAGTGGCACGAAAGACTAAATATTGGTGAGAGACCGATAGCGAACAAGTACTGTGAAGGAAAGATGAAAAGCACTTTGAAAAGAGAGTGAAATAGTACGTGAAATTGTTAATATGGAAGGATATGAAACTAGACACAGAGTCTTGAGTTCTGCAGTCCCTCGGGGTTGTTTTGTCTCTTGATTCTACACCAGCATCAGTTTTGGCAGGAGGATAAACAGTGAGAAATGTAGCTACTTTTGTAGTGTTATAGTCTTACTGCATACTCTTAGCCGAGGACGTGAGGTCAGCCTGTGTAGAGATCGACTGAGCCGGATACCATGCG

>Seq21 [organism=Papiliotrema laurentii] [225CF_isolated from floral nectar of Ipomoea hederifolia (Convolvulaceae)] D1/D2 LSU rDNA gene partial sequence

CAATAAATTGTATTCCCCTAGTAACGGCGAGTGAACCGGGAATAGCTCAAATTTGAAATCTGGCGTCCTCAGGGCGTCCGAGTTGTAATCTATAGAGGCGTTTTCCGTGCCGGACCGTGTCCAAGTTCCTTGGAACAGGATATCAAAGAGGGTGACAATCCCGTACTTGACACGACGACCGGTGCTCTGTGATACGTCTTCTACGAGTCGAGTTGTTTGGGAATGCAGCTCAAAATGGGTGGTGAGTTCCATCTAAAGCTAAATATTGGCGAGAGACCGATAGCGAACAAGTACCGTGAGGGAAAGATGAAAAGCACTTTGGAAAGAGAGTTAAACAGTACGTGAAATTGTTGAAAGGGAAACGATTGAAGTCAGTCGTGACCGAGAGGCTCAGCCGGCTCTGCCGGTGTATTCCCCTCGGTCGGGTCAACATCAGTTTTGTCCGGTGGATAAGGGCGGTAGGAAGGTGGCACCCTCGGGTGTGTTATAGCCTGCCGTCGCATACATCGGGTGAGACTGAGGAACGCAGCTCGCCTTTATGGCCGGGGTTCGCCCACGTCCGAGCTTAGGATGTTGACATAATGGCTTTAAACGACCCGTCTTGAAACACGGACCATAAGTTCAGCGCGTATTCCTACTTTCTTTGACGTCAGAGTCAAAGGTGCGCAATGGCGGTTGTACACACACTCACAAGCACGGTGTGACACATACTCAAGACTCGTGATCTGGGCGAGCGACTCATAAGTGCTTTTAGGCGCGCCGGGTACCAGTGCGCGAGTCCAGCGCCACTCGTCCTAGACGCAAGGAATGACATTTCATGACATCAACAACCTGCCTTCGTATTCAGAGCAACAGTTCTCAGTCGAACTAGCATCCGCACTCATTCGTGTCGGTATCATCTCCGACGGAACGAAGTC

>Seq22 [organism=Hannaella sinensis] [264CF_isolated from floral nectar of Ipomoea hederifolia (Convolvulaceae)] D1/D2 LSU rDNA gene partial sequence

CATAACAAGGTATTCCCCTAGTAGCGGCGAGCGAACTCGGGAAGAGCTCAAATTTAAAATCTGGCGTCTTTCAGGCGTCCGAGTTGTAATCTACAGAAGTGTTTTCCGTGCCGGACCGTGTCCAAGTCCCTTGGAATAGGGTATCAAAGAGGGTGACAATCCCGTACTTGACACGACAACCGGTGCTCTGTGATACATTTTCTACGAGTCGAGTTGTTTGGGAATGCAGCTCAAAATGGGTGGTAAATTCCATCTAAAGCTAAATATAGGCGAGAGACCGATAGCGAACAAGTACCGTGAGGGAAAGATGAAAAGCACTTTGGAAAGAGAGTTAAACAGTACGTGAAATTGTTGAAAGGGAAACGATTGAAGTCAGTCGTGTCTGTTGGTTTCAGCCTTTACCGGTGTATTACCAGCAGACGGGTCAACATCAGTTTTGGACGGTGGAAAAAGACAGGGAGAACGTGGCACCCTCGGGTGTGTTATAGCTCTCTGTTGCATACACTGTCTGAGACTGAGGAATGCAGCTTGCCTTTATGGCCGGGGTTAGCCCAACGTACAAGCTTAGGATGTTGACATAATGGCTGTAAACGACCCGGTCTTGAAACCG

>Seq23 [organism=Pseudozyma sp.] [249CF_isolated from floral nectar of Ipomoea hederifolia (Convolvulaceae)] D1/D2 LSU rDNA gene partial sequence

GGAAAGATTCACGTTATGGCGGGATCCCCTAGTACGGCGGTGAGCGGTCTAGCCCAACCTTGAAAGCCCCCGCTTCGGAATTTGAAGGTAGTCTCAAGAGGAACGTGCCCTTCTAAGTCTATTGTAAAATGCTTGCAAAGGGGGAGATAACCGCGTGTGATTCAACACCATACATATCTTCCCCCTCCAAAGAGTCGAGTTGTTTTCTAAGACACGAGTTAAGTGGGGAATAAATTCCATCTAAAGCTAAATATTGATCTAAGACCTAAATATTGCAAGGACAGTGATGGAAAACAAGAAAGCACTTTGAAAAGAGAGAAAAAAATTGCAAGAAAATGTAAACAGTACGTTTTTTTTTTTTTAAGCACAACTTTTTGTTGAGAAACGCGTTCTGGGATTCAGCCTAAAAGTTGTTTGAAGTTTGTTCCGGAGTGCTGGTCAACGCTGATACCTCCCCACCCTCAAGAGGCCTGGGAATCTGGCACGCTGGGGGGATGGTTGTAGCCTTCTAGTCTTGACAAGGGCCCCAGACCGAGGACAGCAGCGTACTCGCAAGAGCGGGCCTTCGGGCACCTTTACGCTTAGGGCGTTGGCATAATGGCCCTCTACCACCCGTCTTGAAAACGGGACCCACCATGAAACAGGGGTGGTTTGTAAAAAAAAACCTGTCGAGAGTAAACAACATTCCCCGCCGGAGCGTGTTAAAATATACATCCGTCAGTGCGAAAGCTTTACGCGTTTTAGCTAGGTTCTGTAGCGCCGTAAAACAAAACCCAAAAAGGTCTCTTATGAAAAATCTCTCTTGTGAGTAAAAGACCAAAATAGAGGCGTCGTTCTATGAGCTAAGAGAAGAGGGTTTTCTACACGACTCATCTATACCCGGGGTCAACATATGATTCTCTGTCAAGACCCTTACGAAGCGCGGTGCTA

>Seq24 [organism=Sympodiomycopsis paphiopedili] [325CF_isolated from floral nectar of Ipomoea hederifolia (Convolvulaceae)] D1/D2 LSU rDNA gene partial sequence

ACAATGGATGGGTTCCCCTAGTAACGGCGAGTGAAGCGGGAAGAGCTCAAATCTTGAAAGCTGGTACCTTCGGTGCCCGCGTTGTAATCTCGAGAAGTGTTTTCCGTGCTGGACCATGTACAAGTTCCTTGGAATAGGACGTCATAGAGGGTGAAAATCCCGTACTTGACATGGATGCCCAGTGCTTTGTGATACACTCTCCATGAGTCGAGTTGTTTGGGAATGCAGCTCAAAATGAGTGAAAAATTCCATCTAAAGCTAAATATTGGGGAGAGACCGATAGCGAACAAGTACCGTGAGGGAAAGATGAAAAGCACTTTGGAAAGAGAGTTAAACAGTACGTGAAATTGTCGATAAGGGAAGCGCTTAAAGTTAGACATGCCTATTGGGATTCAGCCTTGCTTTTGCTTGGTGTATTTCCCGGTGAGCAGGCCAGCATCAGTTTTGGCTGTCGGATAAGGGTTAGAGGAATGTAGCCCTTCGGGGTGTTATAGCCTTTAACTGGATACGGCGGCTGGGACTGAGGAACGCAGCGTGCCTTTAGGGCGGGCAGTCGGTGCACCTTCTACGCTTAATCCGGATGAACTGACGACGTAATGGCTTGAAG

>Seq25 [organism=Ustilago sp.] [228CF_isolated from floral nectar of Ipomoea hederifolia (Convolvulaceae)] D1/D2 LSU rDNA gene partial sequence

CTAACAAGGTATTCCCCTAGTAACGGCGAGTGAAGTAGGGAAGAGCCCAAGATTGAAAGCTGGCGTCTTCGGCGTCCGCATTGTAATCTCAAGAAGTGTTTTCCGTTTCGGACCATGCCTAAGTCTCTTGGAAAAGAGCATCATAGAGGGTGATAATCCCGTACATGGCATGGAGCGCCCGAGACTTTGTGATACGCTTTCTAAGAGTCGAGTTGTTTGGGAATGCAGCTCAAAATGGGTGGTAAATGCCATCTAAGGCTAAATATTGGGGAGAGACCGATAGCGAACAAGTACAGTGATGGAAAGATGAAAAGAACTTTGAAAAGAGAGTTAAACAGTACGTGAAATTGCCAAAAGGGAAGGGTAGGAGGTCAGAGATGCGTTCTGGGATTCAGCCTTGCTTTTGCTTGGTGTTTTTCCCAGATTGCAGGCCAACGTCGGTTTTGGGCGCTGGAGAAGGGTGGAAGGAATGTGGCACCTCTCGGGGTGTGTTATAGCCTTCTACTGGATACAGTGACCGAGACCGAGGACAGCAGCGTACTCGCAAGAGCGGGCCTTCGGGCACCTTTACGCTTAGGAAGTAGGCATAATGAGCCCTACTACCACTGCAG

>Seq26 [organism=Ustilago sparsa] [256CF_isolated from floral nectar of Ipomoea hederifolia (Convolvulaceae)] D1/D2 LSU rDNA gene partial sequence

AACTAAGGTTTCCCCTAGTACGGCGAGTGAAGAGGGCCGAGCCCAAGTTTGAAAGCTGCGCTTCGGTGTCCGAATGTAATCTCAAGAAGTGTTTTCCGTTTCGGACCATGCCTAAGTCTCTTGGAAAAGAGCATCATAGAGGGTGATAATCCCGTACATGGCATGGAGCGCCCGAGACTTTGTGATACGCTTTCTAAGAGTCGAGTTGTTTGGGAATGCAGCTCAAAATGGGTGGTAAATGCCATCTAAGGCTAAATATTGGGGAGAGACCGATAGCGAACAAGTACAGTGATGGAAAGATGAAAAGAACTTTGAAAAGAGAGTTAAACAGTACGTGAAATTGCCAAAAGGGAAGGGTAGGAGGTCAGAGATGCGTTCTGGGATTCAGCCTTGCTTTTGTTTGGTGTTTTTCCCAGATTGCAGGCCAACGTCGGTTTTGGGCGCTGGAGAAGGGTGGAAGGAATGTGGCACCTCTCGGGGTGTGTTATAGCCTTCTACTGGATACAGTGACCGAGACCGAGGACAGCAGCGTACTCGCAAGAGCGGGCCTTCGGGCACCTTTACGCTTAGGGCGTTGGCATAATGGCCCTCTACCACCCGTCTTGAAACCGGAACCAGAAATTGTTGGTTTGGAGGGCTCCTAAAACACGCGCATGTAGAGGAACTATTCTCGGAGGGGTTTAATAATTACATCGGCGCCTCAATGGAGGACTACCCCTGAACTTAGCTTTCAATAAGCGGGGAAAAGAAACATACAAGGTTTCCCTAGAAACCCGGGGAGGAAGGAGAGGCCAAGATTGAAGTTGGCCCTTCGCTCTCGATTGGTCTCTAGAGGTTTTTTACGTTCGGACATAGCTCAAGAACTTTGGAGCG

>Seq27 [organism=Cryptococcus sp. 1] [217CF_isolated from floral nectar of Ipomoea nil (Convolvulaceae)] D1/D2 LSU rDNA gene partial sequence

ACTAGAGGATTCCCTTAGTAACGGCGAGTGAACCGGGATGAGCTCAAATTTAAAATCTGGCGTCCTTCGGGCGTCCGAGTTGTAATCTATAGAGGCGTTTTCCGCGCCGGACCGCGTCCAAGTCCCTTGGAATAGGGTATCAAAGAGGGTGACAATCCCGTACTTGACGCGACGACCGGTGCTCTGTGATACGTTCTCAACGAGTCGAGTTGTTTGGGAATGCAGCTCTAAATGGGAGGTAAATTCCATCTAAGGCTAAATATTGGCGAGAGACCGATAGCGAACAAGTACCGTGAGGGAAAGATGAAAAGCACTTTGGAAAGAGAGTTAAACAGCACGTGAAATTGTTGAAAGGGAAACGATTGAAGTCAGTCGTGTGGAGGGTATTCAGCCGTCTCTGGCGGTGTACTTGCCCTTCACGGGTCAACATCAGTTTGATCCGGTGGAAAAAGGCTGGGGGAAGGTGGCACCCTCGGGTGTGTTATAGCCCCCTGTCACATACACCGGACCAGACTGAGGAACGCAGCTCGCAGCAATGCCGGGGTTCGCCCACGTACGAGCTTAGGATCTTGATCATAATGGCTCTAAACGATCGCATCG

>Seq28 [organism=Saitozyma flava] [207CF_isolated from floral nectar of Ipomoea nil (Convolvulaceae)] D1/D2 LSU rDNA gene partial sequence

AGAACACCTCATGTACATCTCAGGATTTCCCCTAGTAGCGGCGAGCGAAGCGGGAAGAGCTCAAATTTGTAATCTGGCGTCCTCCGGGCGTCCGAGTTGTAATCTATAGAGACGTTTTCCGTGCCGGACCGTGTCCAAGTCCCTTGGAACAGGGTATCAAAGAGGGTGACAATCCCGTACTTGACGCGACGACCGGTGATCTGTGATACGTTTTCTACGAGTCGAGTTGTTTGGGAATGCAGCTCAAAATAGAAGATAAATTCCATCTAAAGCTAAATATAGGCGAGAGACCGATAGCGAACAAGTACCGTGAGGGAAAGATGAAAAGCACTTTGGAAAGAGAGTTAAACAGTATGTGAAATTGTTGAAAGGGAAACGATTGAAGTCAGTCATGTCCATTGGATTCAGCTGGTTCTGCCAGTGTATTTCCTTTGGACGGGTCAACATCAGTTTGAGCCGGCGGATAATGGCAGAGGGAATGTGGCACCCCCGGGTGTGTTATAGCCCTTTGTCGCATACGTCGGCCCAGACTGAGGAATGCAGCTCGCCTTTAGGGCCGGGGTTAGCCCACGTACGAATTTTCCCGCCTTCACGATCACAAGAATAATGCCTTTAAACGACCCGT

>Seq29 [organism=Sporidiobolus ruineniae] [201CF_isolated from floral nectar of Ipomoea nil (Convolvulaceae)] D1/D2 LSU rDNA gene partial sequence

ACAAGCGGGAATTCCCCTAGTTAGCCGGCGAGCGAAGCGGGAAGAGCTCAAATTTATAATCTGGCGCTTTCAGCGTCCGAGTTGTAATCTCTAGAAGTGTTTTCCGCGTTGGACCGCACATAAGTCTGTTGGAATACAGCGGCACAGTGGTGAGACCCCCGTTCACGGTGCGGATGCCCAATGCTTTGTGATACACTTTCGAAGAGTCGAGTTGTTAGGGAATGCAGCTCAAATTGGGTGGTAAATTCCATCTAAAGCTAAATATTGGCGAGAGACCGATAGCGAACAAGTACCGTGAGGGAAAGATGAAAAGCACTTTGGAAAGAGAGTTAACAGTACGTGAAATTGTTGGAAGGGAAACGCTTGAAGTCAGACTTGCTATTCGGGGCTTGCTCCGAGTTGCAGGCCAGCATCAGTTTTTCGGGGCGGAAAATCACGGATTGAAGGTAGCAGTTTCGGCTGTGTTATAGCTTTCCGTTGGATACGTCCTGGGGGACTGAGGAACGCAGTGTGCTTTTAGCGAGGGAATCGGCTACTTTCACACTTATAGGATGCTAACGATGCACAATACTGCG

>Seq30 [organism=Sympodiomycopsis paphiopedili] [218CF_isolated from floral nectar of Ipomoea nil (Convolvulaceae)] D1/D2 LSU rDNA gene partial sequence

CATAACAAGGTATTCCCCTAGTAACGGCGAGTGAAGTCGGGAAGTAGCTCAAATTTGAAAGCTGGTACCTTCGGTGCCCGCGTTGTAATCTCGAGCAAGTGTTTTCCGTGCTGGACCATGTACAAGTTCCTTGGAATAGGACGTCATAGAGGGTGAAAATCCCGTACTTGACATGGATGCCCAGTGCTTTGTGATACACTCTCCACGAGTCGAGTTGTTTGGGAATGCAGCTCAAAATGGGTGGTAAATTCCATCTAAAGCTAAATATTGGGGAGAGACCGATAGCGAACAAGTACCGTGAGGGAAAGATGAAAAGCACTTTGGAAAGAGAGTTAAACAGTACGTGAAATTGTCGAAAGGGAAGCGCTTAAAGTTAGACATGCCTATTGGGATTCAGCCTTGCTTTTGCTTGGTGTATTTCCCGGTGAGCAGGCCAGCATCAGTTTCGGCTGTCGGATAAGGGTTAGAGGAATGTGGCCCTTCGGGGTGTTATAGCCTTTAACTGGATACGGCGGCTGGGACTGAGGAACGCAGCGTGCCTTTATGGCGGGCAGTCGGGCACCTTCACGCTTAGGATGCTGAGCGTAATGGCTGGAAGCGACCCGTTGCTTG

>Seq31 [organism=Ustilago sp.] [180CF_isolated from floral nectar of Ipomoea nil (Convolvulaceae)] D1/D2 LSU rDNA gene partial sequence

CAATGCCTGATTCCCCTAGTAACGGCGAGTGAAGAGGGAAGAGACCAAGATTGAAAGCTGGCGTCTTCGGCGTCCGCAAAGAAATCTCAAGAAGTGTTTTCCGTTTCGGACCATGCCTAAGTCTCTTGGAAAAGAGCATCATAGAGGGTGATAATCCCGTACATGGCATGGAGCGCCCGAGACTTTGTGATACGCTTTCTAAGAGTCGAGTTGTTTGGGAATGCAGCTCAAAATGGGTGGTAAATGCCATCTAAGGCTAAATATTGGGGAGAGACCGATAGCGAACAAGTACAGTGATGGAAAGATGAAAAGAACTTTGAAAAGAGAGTTAAACAGTACGTGAAATTGCCAAAAGGGAAGTGGTAGGAGGTCAGAGATGCGTTCTGGGATTCAGCCTTGCTTTTGCTTGGTGTTTTTCCCAGATTGCAGGCCAACGTCGGTTTTGGGCGCTGGAGAAGGGTGGAAGGAATGTGGCACCTCTCGGGGTGTGTTATAGCCTTCTACTGGATACAGTGACCGAGACCGAGGACAGCAGCGTACTCGCAAGAGCGGGCCTGTCCCCCACCTTTACGCTTAGGGCGTTGACATAATAAAAAAACAATAGAAACT

>Seq32 [organism=Wickerhamiella occidentalis] [182CF_isolated from floral nectar of Ipomoea nil (Convolvulaceae)] D1/D2 LSU rDNA gene partial sequence

CGCTGAAAATTTTATCAATCGCGTGCTTGCCTTAGTAGTGGCGAATGAACCGGCATTAAGTCCAAATTCGAAATCTGCCAGCAATGGCCGAGTTGTAATTTGAAGATGGTAATTCTGTAGAGAGTCTTTGCTCAAGTTTCCTGGAATGGAACGCCGTGGAGGGTGACAGCCCCGTGAGCCACTGACTTAATACGTGTAGAGTACTATCAAAGAGTCGAGTTGTTTGGGAATGCAGCTCAAATCTCGTGGTAAGTGGCACGAAAGACTAAATATTGGTGAGAGACCGATAGCGAACAAGTACTGTGAAGGAAAGATGAAAAGCACTTTGAAAAGAGAGTGAAATAGTACGTGAAATTTTTTTATATTTTTTTGATATGAAACTAGACACAGAGTCTTGAGTTCTGCAGTCCCTCGGGGTTTGTTTTTTCTCTTGATTCTACACCAGCATCAGTTTTGGCAGGAGGATAAACAGTGAGAAATGTAGCTACTTTTGTAGTGTTATAGTCTTACTGCATACTCTTAGCCGGGACTGAGGTCAGCCTTTGTAGGATGCTGGGGTAATGGTTTTATATCACCCGTCTTGAACGCGGGACCCCTAATTGCGGCATTCACTTAAACCTTTGTCGGGGACTAATAAAAGAAACTTGAGGTTGGGAATGCACATCACATCTCGGGGAAGGTGTACAAACGACTCAAGTTGTCATCTCCCACAACTACCAGTGATGTGAAACACATCACACCTTTTTCAAAAGAGATAAGAAAGGTCCTGTACTTTTTCACACCAAGGATTCAACATCGGCACTGAACCTTGGTTTTGGCGTATCCCCCGGGTTTATTGATCTAGATCCACACACCGCTTAGTATTTGGCGTTGATAACAGGGACAAAGGCATCTTTATATGGTTATGCTACCGGACACATGCCGGACGTAGTCAGCTTCTAGGCTGAACGAATTATATACCGCGGT

>Seq33 [organism=Saitozyma flava] [280CF_isolated from floral nectar of Ipomoea triloba (Convolvulaceae)] D1/D2 LSU rDNA gene partial sequence

ACTAACAAGGCATTCCCCTAGTAGCGGCGAGCGAAGCGGGAAGAGCTCAAATTTGTAATCTGGCGTCCTCCGGGCGTCCGAGTTGTAATCTATAGAGACGTTTTCCGTGCCGGACCGTGTCCAAGTCCCTTGGAACAGGGTATCAAAGAGGGTGACAATCCCGTACTTGACACGACGACCGGTGCTCTGTGATACGTTTTCTACGAGTCGAGTTGTTTGGGAATGCAGCTCAAAATGGGTGGTAAATTCCATCTAAAGCTAAATATAGGCGAGAGACCGATAGCGAACAAGTACCGTGAGGGAAAGATGAAAAGCACTTTGGAAAGAGAGTTAAACAGTATGTGAAATTGTTGAAAGGGAAACGATTGAAGTCAGTCATGTCCATTGGATTCAGCTGGTTCTGCCAGTGTATTTCCTTTGGACGGGTCAACATCAGTTTGAGCCGGCGGATAATGGCAGAGGGAATGTGGCACCCCCGGGTGTGTTATAGCCCTTTGTCGCATACGTCGGCCCAGACTGAGGAATGCAGCTCGCCTTTATGGCCGGGGTAAGCCCACGTACGAGCTTAGGATGTTGGATCGAGTAATATCGCTGCG

>Seq34 [organism=Ustilago sp.] [277CF_isolated from floral nectar of Ipomoea triloba (Convolvulaceae)] D1/D2 LSU rDNA gene partial sequence

CCAGGATGGCTTCCCCTAGTCACGGCGAGTGAAGAGGGAGAGCCCAAGCTTGAAAGCTGGCGCTTCGGCGTCCGATTGTAATCTCAAGAAGTGTTTTCCGTTTCGAACCATGCCTAAGTCTCTTGGAAAAGAGCATCATAGAGGGTGATAATCCCGTACATGGCATGGAGCGCCCGAGACTTTGTGATACGCTTTCTAAGAGTCGAGTTGTTTGGGAATGCAGCTCAAAATGGGTGGTAAATGCCATCTAAGGCTAAATATTGGGGAGAGACCGATAGCGAACAAGTACAGTGATGGAAAGATGAAAAGAACTTTGAAAAGAGAGTTAAACAGTACGTGATATTGTCTAAAGGGAAGGGTAGGAGGTCAGAGATGCGTTCTGGGATTCAGCCTTGCTTTTGCTTGGTGTTTTTCCCAGATTGCAGGCCAACGTCGGTTTTGGGCGCTGGAGAAGGGTGGAAGGAATGTGGCACCTCTCGGGGTGTGTTATAGCCTTCTACTGGATACAGTGACCGAGACCGAGGACAGCAGCGTACTCGCAAGAGCGGGCCTTCGGGCACCTTTACGCTTAGGGCGTTGGCATAATGGCCCTCTACCACCCGTCTTGAAACGGAACCAACCATTTTTTTGGGAGAGGGCCCATGAAAGCCCCCTCATATAGAGAAACAATTCTCGGAAGGGTTTTTATCACTTACCATCGCGCCTCTAGATAGGGAGGACCAACCCCCTAGCTTTAGCGTCTCAAATAAACGGAGGGAAAGAAACTCTACAAGGGTTTTCCTTATGAACAGGCCGTAGAAGGAAGAGCCAAGATGGAATCGGGCTTCCGCGTCAAATGTTACCAGAGTTTGTCGCTCGACATGCCAGAACCTTGCAAAGCATACCAGCCTGTATACAGACTGATGGAGACCCTAGACTCTGTAACCGCTTCCAGAGT

>Seq35 [organism=Pseudozyma sp.] [286CF_isolated from floral nectar of Ipomoea triloba (Convolvulaceae)] D1/D2 LSU rDNA gene partial sequence

ATAGGTGGCTTCCCCTAGTAACTGCGAGTGAAGAGGTTTGAGCCCAACTTCGAAATCTGGCGCTTCGGCGTTGGAGTTGTGATTTAGGGAGGCTTTTCCCGTGCTGGGCAGGGTTACGAGACGGGGGTGAAGGACCATAAGGGGCAATAATCCCGTACCCGGCATGGAGCGCCGGAATGTTTGTGATACCCTTTCTAAGAGTCGAGTTGGTTGGAAATGCAGCTCAATATTGGCGAGAGATGCAATCTAAAGGCTAAATATTGGGGGAGAGACCAATAGCGAACAAGTACAGAGATGAAAAGATGCAAAAGAACTGTGAAAAGAGAGGTAAACAGTACGTGAATTTTTTTTAAAGGGAAGCGGTAGGAGGTCAGAGCATGCCGTCTGGCATTCAGCCTTGCTTTTTGTATTGGTGTTGTTCCCAGTTGGCAGGCAAACGTCGTTTTTGCACACTTGCTGGAAGACTGGGGACGGAATGTGGCACCCCTCGGGTGTGGTTATAGCCTCCTACTGATACAGCGACCGAGACCGAGGACAGCAGCGTACTCGCAAGAGCGGGCCTTCGGCACCTTTACGCTTAGGGCGTTGGCATAATGGCCCTCTACCACCCGTCTTGAAACGGACCCAGCGCGAAAAACGCTTCTCTAATTACACTCGGACGCCGAAGACGCCAGATTTTAATTTGAGCTCATCCCGCTCACTCGCAGTTACTAGGGGAAACCGTTGTTAGTTACGTTTCCTCCGGTTATGACT

>Seq36 [organism=Metschnikowia sp.] [2CF_isolated from floral nectar of Lonchocarpus longistylus (Fabaceae)] D1/D2 LSU rDNA gene partial sequence

ACCAACAGGGATAATGCCTCACTAACGGCGAGTGAAGCGGCAAAAGCTCAAATTTGAAATCCTCCGGGAATTGTAATTTGAAGGCGGGGTTGAATAGGTCTTAGATACTTTAAGTCCATTGGAAAAAGGCGCCATGGAGGGTGATAGCCCCGTAAAAGTATTCAAACCTTCTTTTCTTCCCCTCCTAAAAGTCAAGTTGTTTGGGAATGCACCTCTAAGTGGGTGGTAAATTCCATCTAAAGCTAAATATTGGCAAGAGACCGATAGCGAACAAGTACAGTGATGGAAAGATAAAAAGCACTTTGAAAAGAGAGTGAAAAAGTACGTGAAATTGTTGAAAGGGAAGGGCTTGCAAGCAAACACAACCTCGGTTGGGCCAGCATCGGAGTGGGGGGAGACAAAAAAGGTTAGGAATGTACCTCATCTCAAGTATTATATCCTGGCCCTATATCTCCACCCCCATTCCAAGGCCTGCGATTCTTCAAGGATGCTGGCGTAATGATTGCAAGTCTAAAGG

>Seq37 [organism=Candida versatilis] [58CF_isolated from floral nectar of Malvaviscus arboreus (Malvaceae)] D1/D2 LSU rDNA gene partial sequence

AAACCCACCGGGGTTGCCCTAGTAACGGCGAGTGAAGCGGCAAAAGCCCAAATTTGAAATCCTCTTTTGAGGAGTTGTAATTTGGAGATGGAGTGCTGGACTCAGCTCTGTGGAAGTTGGCTGGAAAGCCGCGCCTTGGAGGGTGATAGCCCCGTGCCACGGAGGTCCTGTGTCTGTGTAGTACTCTTTCTACGAGTCGCGTTGTTTGGGAATGCAGCGCTAAGTGGGTGGTAAATTCCATCTAAGGCTAAATATTGGCGAGAGACCGATAGCGAACAAGTACTGTAAAGGAAAGATGAAAAGCACTTTGAAAAGAGAGTGAAATAGCACGTGAAATCGTTGAAGTGGAAGGGCTTAAAGCTACACACCTTCTTCGGAAGGGGCTAACATCAGTTTTGCCGGATGGATAAATGGTAAAGAAAGTGGCATCTTCGGATGTGTTATAGCTTTATCAAATACGTCCCGGCGGGATTGAGGACCGCGCTTTATGCAGGGAGGTGGCCTTATGGGTTTAAGCCAA

>Seq38 [organism=Aureobasidium sp.] [221CF_isolated from floral nectar of Merremia aegyptia (Convolvulaceae)] D1/D2 LSU rDNA gene partial sequence

ACATGTTGGATCCTGCCCTAGTAACGGCGAGTGAAGCGGCAACTAGCTCAAATCTGAAAGCTGGCCTTCGGGTCCGCATTGTAATTTGTAGAGGATGCTTTGGGGCAGCCGCCTGTCTAAGTTCCTTGGAACAGGACGTCATAGAGGGTGAGAATCCCGTATGTGACAGGACATGGCACCCTATGTAAAGCTCCTTCGACGAGTCGAGTAGAAAAAAGAAAGCAGCTCTAAATGGGAGGTAAATTTCTTCTAAAGCTAAATACCGGCGAGAGACCGATAGCGCACAAGTAGAGTGATCGAAAGATGAAAAGCACTTTGGAAAGAGAGTTAAAAAGCACGTGAAATTGTTGAAAGGGAAGCGCTTGCAATCAGACTTGTTTTGACTGTTCGGCCGGTCTTCTGACCGGTTTACTCAGTCTGGACAGGCCAGCATCAGTTTTGGCGGCCGGATAAAGGCTCAGGGAATGTGGCTCTCACTTCGGTGGGAGTGTTATAGCCCTGGGTGTAATACGGCCAGCCGGGACTGAGTGTGCCGCGCTTCGAGCTAGGAGGTGAACTGAGACCGTAATGGTTGTAAGCG

>Seq39 [organism=Papiliotrema nemorosus] [208CF_isolated from floral nectar of Merremia aegyptia (Convolvulaceae)] D1/D2 LSU rDNA gene partial sequence

AACTAAGCAAGGAATTCCCCCTAGTTAACGGCGAGTTGAACCGGGAAGAGCTCAAATTTGAAATCTGGCGTGCTCAGTGCGTCCGAGTTGTAATCTAAAGAGGCGTTTTCCGTGCCGGACCGTGTCCAAGTCCCTTGGAACAGGGTATCAAAGAGGGTGACAATCCCGTACTTGACACGACGACCGGTGCTCTGTGATACGTCTTCTACGAGTCGAGTTGTCTGGGAATGCAGCTCAAAATGGGTGAGCAAGTTCCATCTAAAGCTAAATATTAGCGAGAGACCGATAGCGAACAAGTACCGTGAGGGAAAGATGAAAAGCACTTTGGAAAGAGAGTTAAACAGTACGTGAAATTGTTAAAAGGGAAACGATTGAAGTCAGTCGTGACCGAGAGGCTCAGCCGGCTCTGCCGGTGTATTCCCCTCGGTCGGGTCAACATCAGTTTTGTCCGGTGGATAAGGACGTCAGGAACGTGGCACCCTCGGGTGTGTTATAGCCTGGCGTCGCATACATCGGGTGAGACTGAGGAATGCAGCTCGCCTTTATGGCCAGGGTTCGCCCATGTCCGAGCTTAGCCATGTCCACATAATGCGCGTTAAACGACTCCGTCCCATG

>Seq40 [organism=Priceomyces melissophilus] [210CF_isolated from floral nectar of Merremia aegyptia (Convolvulaceae)] D1/D2 LSU rDNA gene partial sequence

ACTGAGGGCACTGCCTTAGTAACGGCGAGTGAAGCGGCAAAAGCTCAAACCCGAAATCTGGCATCTTCGATGTCCGAGTTGTAATTTGAAGATTGAAGGTTCATTGGGTCTTGTCTATGTTCCTTGGAATAGGACATCACAGAGGGTGAGAATCCCGTGCGATGAGATTCCGTATTGTAACCTTCTTTCGACGAGTCGAAAAAAATTGGGAATGCAGCTCTAAGTGGGTGGTAAATTCCATCTAAAGCTAAATATTGGCGAGAGACCGATAGCGAACAAGTACAGTGATGGAAAGATGAAAAGAACTTTGAAAAGAGAGTGAAAAAGTACGTGAAATTGTTGAAAGGGAAGGATATGAGATCAGACTTGGTATTTTGCAACCTTACCTTCGTGGTGGGGCCCCTGCAGTTTACTGGGCCAGCATCAATTTGGATGATGGGATAATGACTCAGGAATGTAGCTTTACTTCGGTGAAGTGTTATAGCCTGTGTTGATACCGTCTATCTAGATTGAGGACTGCGTCTTTTGACAAGGATGCTGGCATAATGATCTTATATCACCCGTGGCCTTGCG

>Seq41 [organism=Sympodiomycopsis paphiopedili] [209CF_isolated from floral nectar of Merremia aegyptia (Convolvulaceae)] D1/D2 LSU rDNA gene partial sequence

CAACGACCCTTCCCCTAGTAACGGCGAGAGAAGCGGGTAAGAGCTCAAATTTGAAAGCTGGTACCTTCGGTGCCCGCGTTTGTAATCTCGAGAAGTGTTTTCCGTGCTGGACCATGTACAAGTTCCTTGGAATAGGACGTCATAGAGGGTGAAAATCCCGTACTTGACATGGATGCCCAGTGCTTTGTGATACACTCTCCACGAGTCGAGTTGTTTGGGAATGCAGCTCAAAATGGGTGGTAAATTCCATCTAAAGCTAAATATTGGGGAGAGACCGATAGCGAACAAGTACCGTGAGGGAAAGATGAAAAGCACTTTGGAAAGAGAGTTAAACAGTACGTGAAATTGTCGAAAGGGAAGCGCTTAAAGTTAGACATGCCTATTGGGATTCAGCCTTGCTTTTGCTTGGTGTATTTCCCGGTGAGCAGGCCAGCATCAGTTTCGGCTGTCGGATAAGGGTTAGAGGAATGTGGCCCTTCGGGGTGTTATAGCCTTTAACTGGATACGGCGGCTGGGACTGAGGAACGCAGCGTGCCTTTATGGCGGGCCTTCGGGCAACCTTCACGCTTAGGATGATGGCGTAATGGCTTTAAGCGACCCG

>Seq42 [organism=Papiliotrema laurentii] [105CF_isolated from floral nectar of Merremia dissecta (Convolvulaceae)] D1/D2 LSU rDNA gene partial sequence

AAACTAACAAGGATTCCCCTAGTAACGGCGAGTGAACCGGGAAGAGCTCAAATTTGAAATCTGGCGTCCTCAGGGCGTCCGAGTTGTAATCTATAGAGGCGTTTTCCGTGCCGGACCGTGTCCAAGTTCCTTGGAACAGGATATCAAAGAGGGTGACAATCCCGTACTTGACACGACGACCGGTGCTCTGTGATACGTCTTCTACGAGTCGAGTTGTTTGGGAATGCAGCTCAAAATGGGTGGTGAGTTCCATCTAAAGCTAAATATTGGCGAGAGACCGATAGCGAACAAGTACCGTGAGGGAAAGATGAAAAGCACTTTGGAAAGAGAGTTAAACAGTACGTGAAATTGTTGAAAGGGAAACGATTGAAGTCAGTCGTGACCGAGAGGCTCAGCCGGCTCTGCCGGTGTATTCCCCTCGGTCGGGTCAACATCAGTTTTGTCCGGTGGATAAGGGCGGTAGGAAGGTGGCACCCTCGGGTGTGTTATAGCCTGCCGTCGCATACATCGGGTGAGACTGAGGAACGCAGCTCGCCTTTATGGCCGGGGTTCGCCCACGTCCGAGCTTAGGATGTTGACATAATGGCTTTAAACGAC

>Seq43 [organism=Cryptococcus sp. 2] [166CF_isolated from floral nectar of Merremia dissecta (Convolvulaceae)] D1/D2 LSU rDNA gene partial sequence

AAACTAACAAGGATTCCCCTAGTAACGGCGAGTGAACCGGGAAGAGCTCAAATTTGAAATCTGGCGTGCTCAGTGCGTCCGAGTTGTAATCTATAGAGGCGTTTTCCGTGCCGGACTGTGTCCAAGTCCCTTGGAACAGGGTATCAAAGAGGGTGATAATCCCGTACTTGACACAATGACCGGTGCTCTGTGATACGTCTTCTACGAGTCGAGTTGTTTGGGAATGCAGCTCAAAATGGGTGGTGAGTTCCATCTAAAGCTAAATATTGGCGAGAGACCGATAGCGAACAAGTACCGTGAGGGAAAGATGAAAAGCACTTTGGAAAGAGAGTTAAACAGTACGTGAAATTGTTGAAAGGGAAACGATTGAAGTCAGTCGTGACTGAGAGGCTCAGCCGGTTCTGCCGGTGTATTCCCCTCAGTCGGGTCAACATCAGTTTTGTTCGGTGGATAAGGGCAGTTGGAAGGTGGCACCCTCGGGTGTGTTATAGCCAGCTGTCGCATACATCGGATGAGACTGAGGAATGCAGCTCGCCTTTATGGCCGGGGTTCGCCCACGTTCGAGCTTAGGATGTTGACATAATGGCTTTAAACGAC

>Seq44 [organism=Cryptococcus sp. 3] [179CF_isolated from floral nectar of Merremia dissecta (Convolvulaceae)] D1/D2 LSU rDNA gene partial sequence

CCAGACATGGTTCCCCTACTAACGGCGAGTGAACCGGGAAGAGCTCAAATTTGAAATCTGGCGTCCTCAGGGCGTCCGAGTTGTAATCTATAGAGGCGTTTTCCGTGCCGGACCGTGTCCAAGTTCCTTGGAACAGGATATCAAAGAGGGTGACAATCCCGTACTTGACACGACGACCGGTGCTCTGTGATACGTCTTCTACGAGTCGAGTTGTTTGGGAATGCAGCTCAAAATGGGTGGTGAGTTCCATCTAAAGCTAAATATTGGCGAGAGACCGATAGCGAACAAGTACCGTGAGGGAAAGATGAAAAGCACTTTGGAAAGAGAGTTAAACAGTTTGTTTTATTGTTTTTTGGGAAACGATTGAAGTCAGTCGTGACCGAGAGGCTCAGCCGGTTCTGCTTTTTTATTCCCCTCGGTCGCGTCAACATCAGTTTTGTCCGGTGGATAAGGGCGGTAGGAAGGTGGCACCCTCGGGTGTGTTATAGCCTATCGTCGCATACATCGGGTGAGACTGAGGAACGCAGCTCGCCTTTATGGCCGGGGTTCGCCCACGTCCGAGCTTAGGATGTTGACATAATGGCTTTAAACGACCCGTCTTGAACGGGACCCAGGGCATTCCAATGGGCGGCGAATATCTTCTGGAGTAGGAGATCAAAACAAGCGGACATACCGTAGTTAGAGCACCTATCCCGGCCAGTGAAATAGAGCAATCTATTATCGCTTTGATTGGTACGGCAGGCTTCAAATTAAGTCATTGAGGCCAGCCTAAGCAACATTTGACGCCAGAGTCAAGTCCACCGTACTGTTAAGCCAGGGATAAAGCATTTCGAAGACGCTTAACAGCCTTTCTTTTAACATGATAGCAAAGGAACAATGAATCCAAAGATATCCAAAGCACGACGTTTGACCTCAAATATTCTCATCGATGGCCTCACCAGCCTTCTATAGCCGGGATACAAAGAGATCGTTAGT

>Seq45 [organism=Hannaella siamensis] [107CF_isolated from floral nectar of Merremia dissecta (Convolvulaceae)] D1/D2 LSU rDNA gene partial sequence

AAACTAACAAGGATTCCCCTAGTAGCGGCGAGCGAACCGGGAAGAGCTCAAATTTAAAATCTGGCGTCTTTCAGGCGTCCGAGTTGTAATCTACAGAAGTGTTTTCCGTGCCGGACCGTGTCCAAGTCCCTTGGAATAGGGTATCAAAGAGGGTGACAATCCCGTACTTGACACGACAACCGGTGCTCTGTGATACACTTTCTACGAGTCGAGTTGTTTGGGAATGCAGCTCAAAATGGGTGGTAAATTCCATCTAAAGCTAAATATAGGCGAGAGACCGATAGCGAACAAGTACCGTGAGGGAAAGATGAAAAGCACTTTGGAAAGAGAGTTAAACAGTACGTGAAATTGTTGAAAGGGAAACGATTGAAGTCAGTCGTGACTGTTGGTTTCAGCTGGTTCTGCCAGTGTATTACCAGCAGTCGGGTCAACATCAGTTTTGAGCGGTGGAAAAAGGTATGGAGAACGTAGCACCTTCGGGTGTGTTATAGCTCCGTATTGCATACACTGCTTGAGACTGAGGAATGCAGCTCGCCTTTATGGCCGGGGTTCGCCCACGTTCGAGCTTAGGATGTTGACATAATGGCTTTAAACGAC

>Seq46 [organism=Papiliotrema rajasthanensis] [169CF_isolated from floral nectar of Merremia dissecta (Convolvulaceae)] D1/D2 LSU rDNA gene partial sequence

AAACTAACAAGGATTCCCCTAGTAACGGCGAGTGAACCGGGAAGAGCTCAAATTTGAAATCTGGCGTCCTCAGGGCGTCCGAGTTGTAATCTATAGAGGCGTTTTCCGTGCCGGACCGTGTCCAAGTTCCTTGGAACAGGATATCAAAGAGGGTGACAATCCCGTACTTGACACGATGACCGGTGCTCTGTGATACGTCTTCTACGAGTCGAGTTGTTTGGGAATGCAGCTCAAAATGGGTGGTGAGTTCCATCTAAAGCTAAATATTGGCGAGAGACCGATAGCGAACAAGTACCGTGAGGGAAAGATGAAAAGCACTTTGGAAAGAGAGTTAAACAGTACGTGAAATTGTTGAAAGGGAAACGATTGAAGTCAGTCGTGACTGAGAGGCTCAGCCGGTTCTGCCGGTGTATTCCCCTCAGTCGGGTCAACATCAGTTTTGTCCGGTGGAAAAGGGCGGTAGGAACGTGGCACCTCCGGGTGTGTTATAGCCTGCCGTCGCATACATCGGGTGAGACTGAGGAATGCAGCTCGCCTTTATGGCCGGGGTTCGCCCACGTCCGAGCTTAGGATGTTGACATAATGGCTTTAAACGAC

>Seq47 [organism=Rhodotorula paludigena] [188CF_isolated from floral nectar of Merremia dissecta (Convolvulaceae)] D1/D2 LSU rDNA gene partial sequence

TCAGAAATCTAATCATCATGCCGATTTCCCCTAGTAGCGGCGAGCGAAGCGGGAAGAGCTCAAATTTATAATCTGGCACTTTCAGTGTCCGAGTTGTAATCTCTAGAAGTGTTTTCCGCGTTGGACCGCACACAAGTCTGTTGGAATACAGCGGCACAGTGGTGACACCCCCGTACACGGTGCGGACGCCCAGCGCTTTGTGATACACTTTCGAAGAGTCGAGTTGTTAGGGAATGCAGCTCAAATTGGGTGGTAAATTCCATCTAAAGCTAAATATTGGCGAGAGACCGATAGCGAACAAGTACCGTGAGGGAAAGATGAAAAGCACTTTGGAAAGAGAGTTAACAGTACGTGAAATTGTTGGAAGGGAAACGCTTGAAGTCAGACTTGCTTGCCGGAGCTTGCTTCGGTTTGCAGGCCAGCATCAGTTTTCCGGGGTGGATAATGGCGGTTAGAAGGTAGCAGCTTCAGCTGTGTTATAGCTTTCCGCTGGATACATCCTGGGGGACTGAGGAACGCAGCGTGCTTTTAGCGGGGGAATAGACCCTCTCGGTTTTACCTCTACGACGCTCAGGATCGCTGGTGAAATGGCTTATCC

>Seq48 [organism=Sporidiobolus ruineniae] [109CF_isolated from floral nectar of Merremia dissecta (Convolvulaceae)] D1/D2 LSU rDNA gene partial sequence

AAACTAACAAGGATTCCCCTAGTAGCGGCGAGCGAAGCGGGAAGAGCTCAAATTTATAATCTGGCGCTTTCAGCGTCCGAGTTGTAATCTCTAGAAGTGTTTTCCGCGTTGGACCGCACATAAGTCTGTTGGAATACAGCGGCACAGTGGTGAGACCCCCGTTCACGGTGCGGATGCCCAATGCTTTGTGATACACTTTCGAAGAGTCGAGTTGTTTGGGAATGCAGCTCAAATTGGGTGGTAAATTCCATCTAAAGCTAAATATTGGCGAGAGACCGATAGCGAACAAGTACCGTGAGGGAAAGATGAAAAGCACTTTGGAAAGAGAGTTAACAGTACGTGAAATTGTTGGAAGGGAAACGCTTGAAGTCAGACTTGCTATTCGGGGCTTGCTCCGAGTTGCAGGCCAGCATCAGTTTTTCGGGGCGGAAAATCACGGATTGAAGGTAGCAGTTTCGGCTGTGTTATAGCTTTCCGTTGGATACGTCCTGGGGGACTGAGGAACGCAGTGTGCTTTTAGCGAGGGCTTCGGCTCTTTCACACTTAGGATGCTGGTGGAATGGCTTTAAACGAC

>Seq49 [organism=Ustilago sp.] [177CF_isolated from floral nectar of Merremia dissecta (Convolvulaceae)] D1/D2 LSU rDNA gene partial sequence

CCTTAATGGCTTCCCCTAGTACGGCGAGTGAAGTGGGCCTTAGCCCAAGCTTGAAAGCTGGCGCTTCGGCGTCTGTTTGTAATCTCAAGAAGTGTTTTCCGTTTCGGCCATGCCTAAGTCTCTTGGAAAAGAGCATCATAGAGGGTGATAATCCCGTACATGGCATGGAGCGCCCGAGACTTTGTGATACGCTTTCTAAGAGTCGAGTTGTTTGGGAATGCAGCTCAAAATGGGTGGTAAATGCCATCTAAGGCTAAATATTGGGGAGAGACCGATAGCGAACAAGTACAGTGATGGAAAGATGAAAAGAACTTTGAAAAGAGAGTTAAACAGTACGTGAATTTGCTTTTTAGGGAAGGGTAGGAGGTCAGAGATGCGTTCTGGGATTCAGCCTTGCTTTTGTTTGGTGTTTTTCCCAGATTGCAGGCCAACGTCGGTTTTGGGCGCTGGAGAAGGGTGGAAGGAATGTGGCACCTCTCGGGGTGTGTTATAGCCTTCTACTGGATACAGTGACCGAGACCGAGGACAGCAGCGTACTCGCAAGAGCGGGCCTTCGGGCACCTTTACGCTTAGGGCGTTGGCATAATGGCCCTCTACCACCCGCTTTGAAACGGACCCAAAGTTAGCGGTAGGCCTGCCAATAAAACCCCGCTTGAGGATCAAAATCCTAGGTGATATTTTCCCGCTCATGCGCGCTCAGAGGACTCCATACCGCAGTCTTTCCGTCCCAATATAACGAGCAAAGCTACTTCCAAGGGCGGACTTTATCAAAGCCTGTAGGTAAGGAGAGCCCCAGATGCGATTCGGCCCTCTGCATCCATGATGCCTAGGATGTCGGAGGCCACGGCATGGCAAAACTCTCAACGATCCGCACTTTAGACAGTGTCGTCGAACGTCGGCTCTTAACCGTCTACTCCAGATAGATTG

>Seq50 [organism=Candida parazyma] [165CF_isolated from floral nectar of Operculina pinnatifida (Convolvulaceae)] D1/D2 LSU rDNA gene partial sequence

AATTCAGGGGCACTGCCTTAGTAGCGGCGAGTGAAGCGGCAAAAGCTCAGATTTGAAATCTGCCTCACGGCCGAGTTGTAATCTGTAGATGGCTACTCTGTAACAAGTCTTTGCTCAAGTTTTCTGGAAAGGAACATCATGGAGGGTGATAATCCCGTGAGGCATTGGCGTCGTTGCGTGTAGAGGGTCATCAAAGAGTCGAGTTGTTTGGGAATGCAGCTCAAAGCGGGTGGTAGACACCATCTAAAGCTAAATATTGGCGAGAGACCGATAGCGAACAAGTACTGTGAAGGAAAGATGAAAAGCACTTTGAAAAGAGAGTGAAAAAGTACGTGAAATTATTGATACAGAAGGATATGCCATTGGACGTTATGTTTGGGCATGCTTGTGCTTCGGCGCAGGTTTAACCCTCTCATTTTACCAGCATCAGTTTCGGCAGGAGGACAAACAACGCGGAATGTAGCTGCTTGCAGTGTTATAGACGTGTTGCATACTCTTTGCTGGGATCTGAGGGCCCGCCATTGCAGGATGCTAGCGTAACGATGGCATATCACCCGGCTTG

>Seq51 [organism=Papiliotrema laurentii] [132CF_isolated from floral nectar of Operculina pinnatifida (Convolvulaceae)] D1/D2 LSU rDNA gene partial sequence

AAACTAACAAGGATTCCCCTAGTAACGGCGAGTGAACCGGGAAGAGCTCAAATTTGAAATCTGGCGTCCTCAGGGCGTCCGAGTTGTAATCTATAGAGGCGTTTTCCGTGCCGGACCGTGTCCAAGTTCCTTGGAACAGGATATCAAAGAGGGTGACAATCCCGTACTTGACACGACGACCGGTGCTCTGTGATACGTCTTCTACGAGTCGAGTTGTTTGGGAATGCAGCTCAAAATGGGTGGTGAGTTCCATCTAAAGCTAAATATTGGCGAGAGACCGATAGCGAACAAGTACCGTGAGGGAAAGATGAAAAGCACTTTGGAAAGAGAGTTAAACAGTACGTGAAATTGTTGAAAGGGAAACGATTGAAGTCAGTCGTGACCGAGAGGCTCAGCCGGCTCTGCCGGTGTATTCCCCTCGGTCGGGTCAACATCAGTTTTGTCCGGTGGATAAGGGCGGTAGGAAGGTGGCACCCTCGGGTGTGTTATAGCCTGCCGTCGCATACATCGGGTGAGACTGAGGAACGCAGCTCGCCTTTATGGCCGGGGTTCGCCCACGTCCGAGCTTAGGATGTTGACATAATGGCTTTAAACGAC

>Seq52 [organism=Hannaella siamensis] [134CF_isolated from floral nectar of Operculina pinnatifida (Convolvulaceae)] D1/D2 LSU rDNA gene partial sequence

AAACTAACAAGGATTCCCCTAGTAGCGGCGAGCGAACCGGGAAGAGCTCAAATTTAAAATCTGGCGTCTTTCAGGCGTCCGAGTTGTAATCTACAGAAGTGTTTTCCGTGCCGGACCGTGTCCAAGTCCCTTGGAATAGGGTATCAAAGAGGGTGACAATCCCGTACTTGACACGACAACCGGTGCTCTGTGATACACTTTCTACGAGTCGAGTTGTTTGGGAATGCAGCTCAAAATGGGTGGTAAATTCCATCTAAAGCTAAATATAGGCGAGAGACCGATAGCGAACAAGTACCGTGAGGGAAAGATGAAAAGCACTTTGGAAAGAGAGTTAAACAGTACGTGAAATTGTTGAAAGGGAAACGATTGAAGTCAGTCGTGACTGTTGGTTTCAGCTGGTTCTGCCAGTGTATTACCAGCAGTCGGGTCAACATCAGTTTTGAGCGGTGGAAAAAGGTATGGAGAACGTAGCACCTTCGGGTGTGTTATAGCTCCGTATTGCATACACTGCTTGAGACTGAGGAATGCAGCTCGCCTTTATGGCCGGGTTCGCCCACGTTCGAGCTTAGGATGTTGACATAATGGCTTTAAACGAC

>Seq53 [organism=Kwoniella mangrovensis] [127CF_isolated from floral nectar of Operculina pinnatifida (Convolvulaceae)] D1/D2 LSU rDNA gene partial sequence

ACTAAGCAAGGAATTCCCTTAGTTAACCGGCGAGCGAACCGGGAAGAGCTCAAATCTGAAATCTGGCGTCCTCCGGGCGTCCGAGTTGTAATCTATAGAAGCGTTTTCCGCGCCGGACCGTGTATAAGTCTCCTGGAACGGAGTATCAAAGAGGGTGACAATCCCGTGCTTTACACGACGACCGGTGCTATGTGATACGTTCTCTACGAGTCGAGTTGTTTGGGAATGCAGCTCAAAACGGATAGTAAACTCCATCTAAAGCTAAATATTGGTGGGAGACCGATAGCGAACAAGTACCGTGAGGGAAAGATGAAAAGCACTTTGGAAAGAGAGTTAAACAGTATGTGAAATTGTTGAAAGGGAAACGATTGAAGTCAGTCGTGTCCATTGGGTTCAGCCGGTCCTGCCGGTCTACTCCCTTTGGACGGGTCAACATCAGTTCTGACCGGCGGATAATGGCACGAGGAACGTGGCACCCTCCGGGGTGTGTTATAGCCTCGCGTCGCATACGCTGGTCGGGACTGAGGAACGCAGCTCGCCTTCATGGCCGGAGTACGCCCACGTACGAGCTTAGGATGTTGACATAATGGCTTTAAACGACTTCTCGTCTTGAG

>Seq54 [organism=Metschnikowia ipomoeae] [161CF_isolated from floral nectar of Operculina pinnatifida (Convolvulaceae)] D1/D2 LSU rDNA gene partial sequence

AAACCAACAGGGATTACCTTAGTAACGGCGAGTGAAGCGGTAAAAGCTCAAATTTGAAATCTTCGGAATTGTAATTTGATGGATAAAATTGCCACGGACAAAAGTTCACTGGAAAGTGACGCCGTAGAGGGTGATAGCCCCGTTTACGTCCACCGGCACTATAATTTTATTCCAAAGAGTCGGGTTGTTTGGGAATGCAGCTCAAAGTGGGTGGTAAATTCCATCTAAAGCTAAATATTGACGAGAGACCGATAGCGAACAAGTACAGTGATGGAAAGATGAAAAGAACTTTGAAAAGAGAGTGAAACAGTACGTGAAATTGTTGAAAGGGAAGGGGAGGGAAGATGTTAAAGGGACTCCTTCTTTAGTAGGGATCCCCCCAAGAGTCTTTCCTCTCGC

>Seq55 [organism=Metschnikowia lachancei] [155CF_isolated from floral nectar of Operculina pinnatifida (Convolvulaceae)] D1/D2 LSU rDNA gene partial sequence

AAACCAACAGGGATTGCCTCAGTAACGGCGAGTGAAGCGGCAAAAGCTCAAATTTGAAATCCTTCGGGAATTGTATTTTGAAGAACCTATAAAAAACTCAAAACTACCTTAAGTCTATTGGAAAATGGCGCCATAGAGGGTGATAGCCCCGTAAAGACTAGTCAAGAGTATACTTTTTAGGTCCAAAGAGTCGAGTTGTTTGGGAATGCAGCTCTAAGTGGGTGGTAAATTCCATCTAAAGCTAAATATTGGCGAGAGACCGATAGCGAACAAGTACAGTGATGGAAAGATGAAAAGCACTTTGAAAAGAGAGTGAAAAAGTACGTGAAATTGTTGAAAGGGAAGGGCTTGCAAGCAGACACAACTTTTGTTGGGCCAGCATCGGGGGAGTGGGAGGCAAAAATGAAAAGAAATGTAGCTTCGGTGTTATAGTCTCTTCTTATACCTCCTCACCCTCCCGAGGCCTGCGTATCTAGGATGCTGGCGTAATGGTTGCAAGTCGC

>Seq56 [organism=Metschnikowia lochheadii] [144CF_isolated from floral nectar of Operculina pinnatifida (Convolvulaceae)] D1/D2 LSU rDNA gene partial sequence

AAACCAACAGGGATTACCTCAGTAACGGCGAGTGAAGCGGTAAAAGCTCAAATTTGAAATCTTCGGAATTGTAATTTGATGGATAAAATTGCCACGGACAAAAGTTCACTGGAAAGTGACGCCGTAGAGGGTGATAGCCCCGTTTACGTCCACCGGCACTATAATTTTATTCCAAAGAGTCGGGTTGTTTGGGAATGCAGCTCAAAGTGGGTGGTAAATTCCATCTAAAGCTAAATATTGACGAGAGACCGATAGCGAACAAGTACAGTGATGGAAAGATGAAAAGAACTTTGAAAAGAGAGTGAAACAGTACGTGAAATTGTTGAAAGGGAAGGGGAGGGAAGATGTTAAAGGGACTCCTTCTTTTGTAGGGATCCCCGCCAAAAAGTCTTTCCTCTCGC

>Seq57 [organism=Metschnikowia sp.] [150CF_isolated from floral nectar of Operculina pinnatifida (Convolvulaceae)] D1/D2 LSU rDNA gene partial sequence

AAACCAACAGGGATTGCCTCAGTAACGGCGAGTGAAGCGGCAAAAGCTCAAATTTGAAATCCTCCGGGAATTGTAATTTGAAGGTGGGGTTGAATAGGTCTAGATACTTTAAGTCCATTGGAAAATGGCGCCATGGAGGGTGATAGCCCCGTAAAAGTATTCAAACCTTCTTTTCTTCCCCTCCTAAGAGTCGAGTTGTTTGGGAATGCAGCTCTAAGTGGGTGGTAAATTCCATCTAAAGCTAAATATTGGCGAGAGACCGATAGCGAACAAGTACAGTGATGGAAAGATGAAAAGCACTTTGAAAAGAGAGTGAAAAAGTACGTGAAATTGTTGAAAGGGAAGGGCTTGCAAGCAGACACAACCTCGGTTGGGCCAGCATCGGAGTGGGGGGAGACAAAAAAGGTTAGGAATGTAGCTCATCTCGAGTATTATATCCTGGCCCTATATCTCCACCCCCTTCCGAGGCCTGCGATTCTTCAAGGATGCTGGCGTAATGGTTGCAAGTCGC

>Seq58 [organism=Rhodotorula paludigena] [185CF_isolated from floral nectar of Operculina pinnatifida (Convolvulaceae)] D1/D2 LSU rDNA gene partial sequence

AACTAAGCAAGGATTCCCCTAGTAGCGGCGAGCGAAGCGGGAAGAGCTCAAATTTATAATCTGGCACTTTCAGTGTCCGAGTTGTAATCTCTAGAAGTGTTTTCCGCGTTGGACCGCACACAAGTCTGTTGGAATACAGCGGCACAGTGGTGACACCCCCGTACACGGTGCGGACGCCCAGCGCTTTGTGATACACTTTCGAAGAGTCGAGAAAAAAAAAGAAAGCAACTCAAATTGGGTGGTAAATTCCATCTAAAGCTAAATATTGGCGAGAGACCGATAGCGAACAAGTACCGTGAGGGAAAGATGAAAAGCACTTTGGAAAGAGAGTTAACAGTACGTGAAATTGTTGGAAGGGAAACGCTTGAAGTCAGACTTGCTTGCCGGAGCTTGCTTCGGTTTGCAGGCCAGCATCAGTTTTCCGGGGTGGATAATGGCGGTTAGAAGGTAGCAGCTTCAGCTGTGTTATAGCTTTCCGCTGGATACATCCTGGGGGACTGAGGAACGCAGCGTGCTTTTAGCGGGGATTTAGACCACTTCACGCTTAGCATGCTCGTCGACGTACTATCGGCTGCTG

>Seq59 [organism=Wickerhamiella occidentalis] [137CF_isolated from floral nectar of Operculina pinnatifida (Convolvulaceae)] D1/D2 LSU rDNA gene partial sequence

AAACCAACAGGGATTGCCTTAGTAGTGGCGAATGAACCGGCAAAAGTCCAAATTTGAAATCTGCCAGCAATGGCCGAGTTGTAATTTGAAGATGGTAATTCTGTAGAGAGTCTTTGCTCAAGTTTCCTGGAATGGAACGCCGTGGAGGGTGACAGCCCCGTGAGGCATTGACTTAATACGTGTAGAGTACTATCAAAGAGTCGAGTTGTTTGGGAATGCAGCTCAAATCTCGTGGTAAGTGGCACGAAAGACTAAATATTGGTGAGAGACCGATAGCGAACAAGTACTGTGAAGGAAAGATGAAAAGCACTTTGAAAAGAGAGTGAAATAGTACGTGAAATTGTTAATATGGAAGGATATGAAACTAGACACAGAGTCTTGAGTTCTGCAGTCCCTCGGGGTTGTTTTGTCTCTTGATTCTACACCAGCATCAGTTTTGGCAGGAGGATAAACAGTGAGAAATGTAGCTACTTTTGTAGTGTTATAGTCTTACTGCATACTCTTAGCCGGGACTGAGGTCAGCCTTTGTAGGATGCTGGGGTAATGGTTTTATATCAC

>Seq60 [organism=Kurtzmaniella cleridarum] [94CF_isolated from floral nectar of Opuntia dillenii (Cactaceae)] D1/D2 LSU rDNA gene partial sequence

ACTCAACAGGCATTGCCTTAGTAACGGCGAGTGAAGCGGCAAAAGCTCAAATTTGAAATCTAGTACTTTCAGTGCTCGAGTTGTAATTTGAAGAAGGTAACTTTGGTGTTGGCCCTTGTCTATGTTCCTTGGAACAGGACGTCACAGAGGGTGAGAATCCCGTGCGATGAGGTGTCCAATACTATGTAAAGTGCTTTCGAAGAGTCGAGTTGTTTGGGAATGCAGCTCTAAGTGGGTGGTAAATTCCATCTAAAGCTAAATATTGGCGAGAGACCGATAGCGAACAAGTACAGTGATGGAAAGATGAAAAGAACTTTGAAAAGAGAGTGAAAAAGTACGTGAAATTGTTGAAAGGGAAGGGTTTGAGATCAGACTTGGTATTTTGCAATCCTTTCCTTCTCGGGGAGGTTTCTTAGCAGCTTACCGGGCCAGCATCGGTTTGGATGCCACCACAACTGACATTGGAATGTAGCTCTTCGGAGTGTTATAGACTTTGTTGATACTGCCTATCTAGACCGAGGACTGCGTCTTTGATCTAGGATGCTGCCGTAATGATCTTAAACCGTCCCGTCTA

>Seq61 [organism=Candida etchellsii] [80CF_isolated from floral nectar of Opuntia dillenii (Cactaceae)] D1/D2 LSU rDNA gene partial sequence

CCGTGCATATCAATAAGCGAGAGGAAAAGAAACCAACAGGGGAGGGAACTAGTAGACGGCGAGTGAACAGGCAAGAGCTCAGATTTGAAAGCCTTTTTGGCATTGTATTCTGAAGTCAGTTTCCTGACTATCGGTGTTTAAGTCTTCTGGAAAGGAGCGCCATGGAGGGTGATAGCCCCGTACGACACCTCACTCATTGTAGGATTCTGACGTGGAGTCGAGTTGTTTGGGAATGCAGCTCAAAAGGGTGGTATGCTCCATCTAAAGCTAAATATCTGTGAGAGACCGATAGCGAACAAGTACTGTGAAGGAAAGATGAAAAGAACTTTGAAAAGAGAGTGAAAAAGTACGTGAAATTGTTGAAATGGAAGGATATGCCGCTAACAATGTAGAGCCGTGTTTGGGGGGAAGACAAAAGCTGAAGAATGTAACTCTTTTAGGGAATTATAACTTCAGTCAATATTCGCCATGCCAAGCGACGAGGACCTGCAGGCTCTACTATTTTAATGGGGAGAAAAT

>Seq62 [organism=Candida bombi] [51CF_isolated from floral nectar of Passiflora foetida (Passifloraceae)] D1/D2 LSU rDNA gene partial sequence

AAACCAACAGGGATTGCCTTAGTAGCGGCGAGTGAACCGGCAAAAGCTCAGATTTGAAAGCCTTTCGGGGCATTGTATTCTGAAGCCTTTATCCTGGGAAACCGATGTCCAAGTCTACTGGAACGTAGTGCCATGGAGGGTGATAGCCCCGTACGACATTGACCCCATTGTAGGATTTTGGCGTGGAGTCGAGTTGTTTGGGAATGCAGCTCAAATGGGTGGTATGCTCCATCTAAAGCTAAATATCTGCGAGAGACCGATAGCGAACAAGTACTGTGAAGGAAAGATGAAAAGAACTTTGAAAAGAGAGTGAAAAAGTACGTGAAATTGTTGAAATGGAAGGGTAGGCCGCTAACCACGTAGAGTCGTGTTTGGGGGGAAGATAAAAGCCAGAGAATGTAACTCCTCGGAGTATTATAGCTCTGGTCAATATTCCCATCCGGGCGCGAGGACCGAAGACTCTGCTAAATGGTGGTCTACCAC

>Seq63 [organism=Candida sorbosivorans] [14CF_isolated from floral nectar of Passiflora foetida (Passifloraceae)] D1/D2 LSU rDNA gene partial sequence

CAGAAATCCAACGCGAGTGTTGATTGCGCCTACTAATGGCGAATGAACAGGCAATAACCTCAGATTTGAAACCCTCGGGATTGTAATCTGGAGTACCCGGATTTGGCACGCTGACCAAGTCTTCTGGAACGGAGCGCCATGGAGGGTGACAGCCCCGTAGCACCAGCCACTGTAAATCCGGGTCGACGAGTCGAGTTGTTTGGGAATGCAGCTCTAAGTGGGTGGTATGCTCCATCTAAAGCTAAATATTGGCGAGAGACCGATAGCGAACAAGTACTGTGAAGGAAAGATGAAAAGAACTTTGAAAAGAGAGTGAAAAAGTACGTGAAATTGTTGAAATGGAAGGCAATGAGGTGCGATTGAACCGGACGTTTGCGGGCAGGACAAAAGCGCAGGCCGCCTCGGCATTGCCTGCGTGCATACTGCCTCGCGGACTACCGGTACTAACAACGCCTAGAGAAATGCACCCGTAATGAA

>Seq64 [organism=Vishniacozyma taibaiensis] [42CF_isolated from floral nectar of Piscidia piscipula (Fabaceae)] D1/D2 LSU rDNA gene partial sequence

CTAACGTACGACTCCCTTAGTAACGGCGAGCGAACCGGGATGAGCTCAAATTTAAAATCTGGCGTCTTTCAGGCGTCCGAGCTGTAATCTATAGAGGCGTTTTCCGCGCCGGACCGCGTCCAAGTCCCTTGGAATAGGGTATCAAAGAGGGTGACAATCCCGTACTTGACGCGACAACCGGTGCTCTGTGATACGTTCTCAACGAGTCGAGTTGTTTGGGAATGCAGCTCTAAATGGGTGGTAAATTCCATCTAAGGCTAAATATTGGCGAGAGACCGATAGCGAACAAGTACCGTGAGGGAAAGATGAAAAGCACTTTGGAAAGAGAGTTAAACAGCACGTGAAATTGTTAAAAGGGAAACGATTGAAGTCAGTCGTGTGGGAGGTATTCAGCCGTCTCTGGCGGTGTATTTGCCTCTCACGGGTCAACATCAGTTTGATTCGGTGGAAAAAGGCGGGAGGAAGGTGGCACCCTCGGGTGTGTTATAGCCTCCTGTCATATACACTGGACCAGACTGAGGAACGCAGCTCGCAGCAATGCCGGGGTTCGCCCAACGTACGAGCTTAGGATGTTGACATAAGATAGAGCTGTT

>Seq65 [organism=Naganishia liquefaciens] [46CF_isolated from floral nectar of Piscidia piscipula (Fabaceae)] D1/D2 LSU rDNA gene partial sequence

CCGTACAACGATTCCCCTAGTAACGGCGAGTGAAGCGGGAACAGCTCAAATTTGAAATCTAGTAGCCTTCGGCTGCTCGAGTTGTAATCTAGAGAAGTGTTTTCCGTGCCGGCCCATGTACAAGTCCCTTGGAACAGGGCGTCATAGAGGGTGAGAATCCCGTCCTTGACATGGACCCCCGGTGCTCTGTGATACACTTTCAACGAGTCGAGTTGTATGAGAATGCAGCTCAAAATGGGTGGTGAATTCCATCTAAAGCTAAATATTGGCGAGAGACCGATAGCGAACAAGTACCGTGAGGGAAAGATGAAAAGCACTTTGGAAAGAGAGTTAAACAGTACGTGAAATTGTTGAAAGGGAAACGATTGAAGTCAGTCATGCTCTTTGGTATTTATATCATTGAGTGGGGTCAACATCAGTTTTGATCGATGGATAAAGGCACTAGGAAGGTAGCACTCTCGGGTGAACTTATAGCCAAGCGTCATATACATTGATTGGGACTGAGGAACGCAGCATGCCTTTATGGCCGGGATTCGTCCACGTACATGCTTAGGATGTTGACATACTACAATCGTGCTTTAAACGT

>Seq66 [organism=Sympodiomycopsis paphiopedili] [48CF_isolated from floral nectar of Piscidia piscipula (Fabaceae)] D1/D2 LSU rDNA gene partial sequence

CTAACAAGGCATTCCCCTAGTAACGGCGAGTGAAGCGGGAAGAGCTCAAATTTGAAAGCTGGTACCTTCGGTGCCCGCGTTGTAATCTCGAGAAGTGTTTTCCGTGCTGGACCATGTACAAGTTCCTTGGAATAGGACGTCATAGAGGGTGAAAATCCCGTACTTGACATGGATGCCCAGTGCTTTGTGATACACTCTCCACGAGTCGAGTTGTTTGGGAATGCAGCTCAAAATGGGTGGTAAATTCCATCTAAAGCTAAATATTGGGGAGAGACCGATAGCGAACAAGTACCGTGAGGGAAAGATGAAAAGCACTTTGGAAAGAGAGTTAAACAGTACGTGAAATTGTCGAAAGGGAAGCGCTTAAAGTTAGACATGCCTATTGGGATTCAGCCTTGCTTTTGCTTGGTGTATTTCCCGGTGAGCAGGCCAGCATCAGTTTTGGCTGTCGGATAAGGGTTGGAGGAATGTGGCCCCTCGGGGTGTTATAGCCTCTAACTGGATACGGCGGCCGGGACTGAGGAACGCAGCGTGCCTTTATGGCGGGCCTTCGGGCACCTTCACGCTTAGGATGCTGGCGTAATAGCTTTAAGCGACCAGT

>Seq67 [organism=Metschnikowia koreensis] [36CF_isolated from floral nectar of Tecoma stans (Bignoniaceae)] D1/D2 LSU rDNA gene partial sequence

AAACCAACAGGGATTACCTTAGTAACGGCGAGTGAAGCGGTAAAAGCTCAAATTTGAAATCTTCGGAATTGTAATTTGATGGATAAAATTGCCACGGACAAAAGTTCACTGGAAAGTGACGCCGTAGAGGGTGATAGCCCCGTTTACGTCCACCGGCACTATAATTTTATTCCAAAGAGTCGGGTTGTTTGGGAATGCAGCTCAAAGTGGGTGGTAAATTCCATCTAAAGCTAAATATTGACGAGAGACCGATAGCGAACAAGTACAGTGATGGAAAGATGAAAAGAACTTTGAAAAGAGAGTGAAACAGTACGTGAAATTGTTGAAAGGGAAGGGGAGGGAAGATGTTAAAGGGACTCCTTCTTTAGTAGGGATCCCCCCAAGAGTCTTTCCTCTCGC

>Seq68 [organism=Metschnikowia ipomoeae] [27CF_isolated from floral nectar of Tecoma stans (Bignoniaceae)] D1/D2 LSU rDNA gene partial sequence

AAACCAACAGGGATTGCCTCAGTAACGGCGAGTGAAGCGGCAAAAGCTCAAATTTGAAATCCTCCGGGAATTGTAATTTGAAGGTGGGGTTGAATAGGTCTAGATACTTTAAGTCCATTGGAAAATGGCGCCATGGAGGGTGATAGCCCCGTAAAAGTATTCAAACCTTCTTTTCTTCCCCTCCTAAGAGTCGAGTTGTTTGGGAATGCAGCTCTAAGTGGGTGGTAAATTCCATCTAAAGCTAAATATTGGCGAGAGACCGATAGCGAACAAGTACAGTGATGGAAAGATGAAAAGCACTTTGAAAAGAGAGTGAAAAAGTACGTGAAATTGTTGAAAGGGAAGGGCTTGCAAGCAGACACAACCTCGGTTGGGCCAGCATCGGAGTGGGGGGAGACAAAAAAGGTTAGGAATGTAGCTCCCTTTAGAGTATTATATCCTAGCCCTATATCTCCGTCCCCCTTCCTAGGCCGGCGATTCTTCAAGGATGCTGGCGTAATGGTTGCAAGTCTCCTGTCC

>Seq69 [organism=Cryptococcus sp. 2] [24CF_isolated from floral nectar of Tecoma stans (Bignoniaceae)] D1/D2 LSU rDNA gene partial sequence

AAACTAACAAGGATTCCCCTAGTAACGGCGAGTGAACCGGGAAGAGCTCAAATTTGAAATCTGGCGTGCTCAGTGCGTCCGAGTTGTAATCTATAGAGGCGTTTTCCGTGCCGGACTGTGTCCAAGTCCCTTGGAACAGGGTATCAAAGAGGGTGATAATCCCGTACTTGACACAATGACCGGTGCTCTGTGATACGTCTTCTACGAGTCGAGTTGTTTGGGAATGCAGCTCAAAATGGGTGGTGAGTTCCATCTAAAGCTAAATATTGGCGAGAGACCGATAGCGAACAAGTACCGTGAGGGAAAGATGAAAAGCACTTTGGAAAGAGAGTTAAACAGTACGTGAAATTGTTGAAAGGGAAACGATTGAAGTCAGTCGTGACTGAGAGGCTCAGCCGGTTCTGCCGGTGTATTCCCCTCAGTCGGGTCAACATCAGTTTTGTTCGGTGGATAAGGGCAGTTGGAAGGTGGCACCCTCGGGTGTGTTATAGCCAGCTGTCGCATACATCGGATGAGACTGAGGAATGCAGCTCGCCTTTATGGCCGGGGTTCGCCCACGTTCGAGCTTAGGATGTTGACATAATGGCTTTAAACGAC
